# Bioinspired Suspended Sensing Membrane Array with Modulable Wedged‐Conductive Channels for Crosstalk‐Free and High‐Resolution Detection

**DOI:** 10.1002/advs.202403645

**Published:** 2024-05-08

**Authors:** Haixuan Luo, Xiaoliang Chen, Sheng Li, Jinbin Xu, Xiangming Li, Hongmiao Tian, Chunhui Wang, Bo Li, Manman Zhang, Bai Sun, Juan He, Jinyou Shao

**Affiliations:** ^1^ State Key Laboratory for Manufacturing Systems Engineering Xi'an Jiaotong University Xi'an Shaanxi 710049 China; ^2^ Frontier Institute of Science and Technology (FIST) Xi'an Jiaotong University Xi'an Shaanxi 710049 China; ^3^ Department of Rehabilitation First Affiliated Hospital of Xi'an Jiaotong University Xi'an Shaanxi 710061 China

**Keywords:** bioinspired, crosstalk‐free, customized structures, high density, microchannels, sensor array

## Abstract

High spatial‐resolution detection is essential for biomedical applications and human‐machine interaction. However, as the sensor array density increases, the miniaturization will lead to interference between adjacent units and deterioration in sensing performance. Here, inspired by the cochlea's sensing structure, a high‐density flexible pressure sensor array featuring with suspended sensing membrane with sensitivity‐enhanced customized channels is presented for crosstalk‐free and high‐resolution detection. By imitating the basilar membrane attached to spiral ligaments, a sensing membrane is fixed onto a high‐stiffness substrate with cavities, forming a stable braced isolation to provide an excellent crosstalk‐free capability (crosstalk coefficient: 47.24 dB) with high‐density integration (100 units within 1 cm^2^). Similar to the opening of ion channels in hair cells, the wedge‐type expansion of the embedded cracks introduced by stress concentration structures enables a high sensitivity (0.19 kPa^−1^) and a large measuring range (400 kPa). Finally, it demonstrates promising applications in distributed displays and the condition monitoring of medical‐surgical intubation.

## Graphical abstract

The high‐performance suspended pressure sensor array based on modulable wedged‐conductive channels allows high‐density integration (100 units within 1 cm^2^) with the help of high‐precision constraint printing. The sensing performance can be regulated with customized sensitive structures and more importantly, the array can achieve excellent crosstalk‐free effect under the support of high‐stiffness substrate.

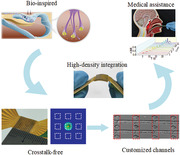

## 1 Introduction

With the increasing advancement of distributed display and dynamic detection, it has been seen extensive usage of high‐density flexible sensor arrays in medical treatment,^[^ ^1^, ^2^, ^3^, ^4^, ^5^ ^]^ man‐machine interaction,^[^ ^6^, ^7^, ^8^ ^]^ robotic technology,^[^ ^9^, ^10^, ^11^, ^12^ ^]^ and mechanical fault diagnosis.^[^ ^13^, ^14^, ^15^, ^16^ ^]^ For example, the tiny size makes it easier to integrate the part of the surgical instrument that contacts the tissue, which can monitor the pressure distribution during surgery in real time to avoid excessive pressure damage to the tissue.^[^ ^17^, ^18^ ^]^ It has been witnessed that the flexible sensor array with a high‐density property owns good adaptability and is easy to apply to various curved objects to achieve accurate data acquisition.^[^ ^19^, ^20^, ^21^ ^]^ The dense distribution provides simultaneous multi‐pixel perception within a small area, facilitating easier integration into various systems and enhancing performance and reliability. However, with the decrease in unit size, the challenges of the crosstalk effect and reduced sensing performance of high‐density flexible pressure sensor arrays become more prominent. The crosstalk phenomenon is mainly induced by a mechanical structure of distributed design and electrical signal transmission.^[^ ^22^, ^23^ ^]^ In addition, the miniaturization of the unit size brings new difficulties to the design and manufacture of sensitive structures.^[^ ^24^, ^25^ ^]^ How to obtain high sensitivity and a wide range of sensing performance under the premise of reducing crosstalk is an urgent problem to be solved.

A high‐density flexible sensor array is particularly susceptible to buy signal interference between adjacent sensors (crosstalk) due to its high integration and dense layout, leading to inaccuracies in data collection.^[^ ^26^, ^27^ ^]^ These disturbances arise from the diffusion of mechanical strain^[^ ^22^, ^28^, ^29^ ^]^ and electrical signals generated by common conduction and electromagnetic induction.^[^ ^23^, ^30^, ^31^ ^]^ Nowadays, there are two main strategies to decrease the crosstalk in a high‐density sensor array. The first strategy involves using the back‐end processing circuit or the arithmetic method to fade out crosstalk,^[^ ^32^ ^]^ but the underlying mechanical interactions still cannot be ignored and it is an incomplete method of eliminating crosstalk. The second way is to decrease crosstalk from sensor structure design radically. Kim et al.^[^ ^22^ ^]^ used the mesh template to prepare an electrically isolated piezoresistive composite array to suppress the crosstalk of adjacent sensing units, but only 180 mm × 100 mm expandable array area can be obtained due to the limitation of the mesh template size. Zhao et al.^[^ ^17^ ^]^ used a laser manufacturing process to carbonize polyimide film and cut it into separate sensing units connected by serpentine structures to reduce the strain transfer of adjacent units, and obtained a pressure sensing array with a unit size of 0.7 mm. In conclusion, addressing crosstalk through structural design is a fundamentally effective approach to mitigate signal interference. However, further research and optimization in the structure and manufacturing methods to achieve the high‐precision and efficient production of high‐density sensor arrays are still essential.

Another limiting factor of the high‐density sensor array is the weakened performance produced by the small unit size. As the size of individual sensing units decreases, the signal generated by each unit tends to be weaker compared to the noise present in the system. For general sensing arrays, sandwiching a polymer dielectric or conductive layer between two electrode arrays is a common and simple construction strategy, yet it suffers from low sensitivity.^[^ ^33^, ^34^, ^35^, ^36^ ^]^ Furthermore, some strategies that reflect pressure changes through the deformation of 3D microstructures are proposed to enhance performance, such as micro‐pyramids,^[^ ^37^, ^38^ ^]^ micro‐domes,^[^ ^24^, ^39^ ^]^ micro‐pillars,^[^ ^40^, ^41^, ^42^ ^]^ etc. Obviously, the design of these microstructures can improve the sensitivity of the sensor array, but the pressure measurement range is largely sacrificed due to the reduction of equivalent stiffness. To overcome the trade‐off between sensitivity and measurement range, the researchers proposed hierarchical composite structure constructions,^[^ ^43^, ^44^, ^45^ ^]^ using them to deform step by step under pressure, while improving the sensing performance. However, the hierarchical structures involved are intricate, and their manufacturing processes are quite laborious, which hinders the miniaturization of the sensor unit. Additionally, maintaining structural uniformity in high‐density applications is challenging, posing a significant hurdle in developing a viable high‐density array that preserves the integrity of various sensing parameters.

The basal membrane of vibration within the cochlea of the human ear can transform mechanical vibrations into electroneurographic signals. It has a sensitive property due to the noticeable changes in ion channels and the ample flexibility provided by the cochlear duct. At the same time, the basal membrane is supported by high‐stiffness spiral ligaments, which could shield it from unwanted disturbances. Herein, inspired by the physiological structures and sensing behaviors of cochlea, we propose a high‐density crosstalk‐free sensor array based on a customized channel sensing membrane. This sensor array integrates an upper suspended sensitive membrane, the electrode layer with copper electrodes on a polyimide (PI) film as well as a high elastic modulus support layer with spacing cavities, forming a braced isolation sensing structure. Under external pressure, only the suspended unit area above the cavity undergoes significant strain, and the strain diffusion is eliminated between adjacent units under the support of the structured high‐stiffness substrate, so as to achieve the effect of mutual isolation of the unit signals (crosstalk coefficients of resistance change rate: 47.24 dB). Moreover, the customized ion channel‐type and wedge‐like cracks are created within flexible membrane constraints by microchannels and stress concentration structures to achieve both high sensitivity and robust stability, which can be used to control the sensing performance and achieve better consistency. Finally, a high‐density sensor array (100 units within 1 cm^2^) is achieved, with a wide pressure measurement range of 400 kPa, a maximum sensitivity of 0.19 kPa^−1^, and excellent cycle stability (10 000 cycles). The prepared sensor array has been demonstrated to be useful in the applications of pressure distribution display and auxiliary intubation operation in the medical field.

## 2 Results and Discussion

### 2.1 Inspiration and Mechanism of the Crosstalk‐Free Suspended Sensing Membrane Array

**Figure 1:**
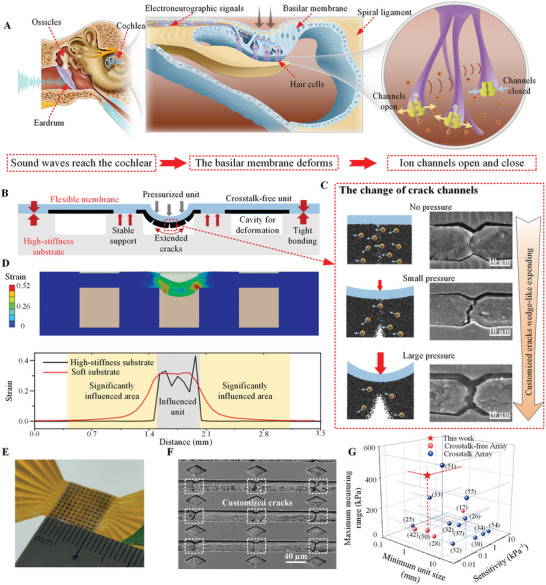
Inspiration and mechanism of the crosstalk‐free suspended sensing membrane array. A) The structure and perceptual mechanisms of the human ear and cochlea. B) The crosstalk‐free design of the high‐density sensor array. C) The sensing mechanism of the crack expansion in the upper membrane under different pressures. D) The simulation and compression strain curves of the crosstalk‐free effectiveness of a flexible membrane on a high‐stiffness substrate and a soft support, respectively. E) The high‐density crosstalk‐free sensor array sample. F) The SEM image of customized cracks controlled by the structured PDMS template. G) Comparisons of performances and dimensional characteristics of flexible sensor arrays in different research.

**Figure** **1**A shows the sensing process by which the human cochlea converts mechanical movements into electroneurographic signals and points to key components of the auditory system. Sound waves first travel through the inner ear through structures such as the eardrum and ossicles, thereby causing mechanical vibrations in the cochlea, which facilitate the conversion to nerve impulses. Within the internal structure of the cochlea, the sensitive and suspended basilar membrane is deformed by the varying pressure from lymphatic fluid, caused by sound waves.^[^ ^46^ ^]^ There are many tiny hair cells with ion channels that can open and close distributed on the basilar membrane, which are the units involved in signal transformation. These hair cells will be relatively skewed with the deformation of the basilar membrane, resulting in changes in the closure of ion channels on them. This allows potassium ions from the lymphatic fluid to enter the open hair cells, while the change in concentration triggers the opening of gates on the opposite side for calcium ions transfer. This ion exchange then triggers the actual impulses in the central nervous system, which achieves the conversion from mechanical signals to electrical signals.^[^ ^47^ ^]^ At the same time, the spiral ligaments,^[^ ^48^ ^]^ which are characterized by high stiffness compared with other soft tissues, keep the basilar membrane in the right position and the cochlea structure stable. It also acts as a buffer, absorbing the energy from excessively loud sounds and protecting the fragile structures of the inner ear. Overall, the function of this connective tissue provides a critical strategy for ensuring the stability and isolation of the sensing structure.

The sensing mechanism of the cochlea inspires the construction strategy for a high‐performance, crosstalk‐free sensor array. Here, a sensing array with sensitivity‐enhanced customized cracks like the cochlea structure is shown in Figure 1B, consisting of an easily deformed polydimethylsiloxane (PDMS) membrane embedded with carbon nanotube (CNT) conductive channels and a high‐stiffness support isolation structure. The upper membrane can transmit mechanical deformation and convert it into measured signals, similar to the basilar membrane. This function is triggered by the opening and closing of customized CNT sensing cracks with the same structural change of ion channels to realize the conversion of electrical signals. The high‐stiffness substrate, a type of light‐curable material, possesses a higher elastic modulus^[^ ^49^ ^]^ (137.9 MPa) and tensile strength (10.34 MPa) than the upper membrane, ensuring minimal deformation outside the pressurized area. This substrate features a Shore hardness of 50 HD and can withstand 80% of its ultimate tensile strength so that it can provide stable rigid support. Consequently, the central unit is influenced evidently while other units do not deform under the action of the combination of the flexible sensing membrane and the structured high‐stiffness substrate. Figure 1C shows the deformation details of customized CNT sensing cracks and verified SEM images. After the sensing membrane is stretched and then returned to its original state, customized CNT cracks are formed within the PDMS template. At this time, cracks do not break completely and most of the broken section is still connected, leading to good electrical conductivity in microchannels. As the pressure is applied and increases, the sensing membrane generates strain, causing wedge‐shaped cracks to emerge on the surface. These cracks develop gradually due to the thickness of the CNT confined within the microchannels. The resistance of the CNT channel also increases due to the gradual narrowing of the electron conduction path. Finally, this expanding property of the conductive material enhances sensitivity and widens the measurement range. Figure 1D demonstrates the strain simulation of an upper flexible membrane with high‐stiffness substrate support. As 200 kPa pressure is applied to the central unit, the membrane on the cavity appears strained, especially at the contact area with bottom support. On the contrary, adjacent units are not influenced and show no strain, proving the effectiveness of crosstalk‐free with cavity rigid support structure. Figure S1 (Supporting Information) shows the result of applying 0, 20, 80, 160, 240, 320, and 400 kPa pressure on the same single unit respectively, substantiating that the strain of the upper membrane increases gradually with the increase of pressure. We also compared the aforementioned design with the same integrated structure, which uses the soft material (Ecoflex) as a bottom substrate. Strain curves show there is a larger significantly influenced area on the soft substrate when applying the same pressure, indicating the poor crosstalk‐free property. Besides, strain curves from 0°, 45°, and 90° directions respectively in an array of 3 × 3 units are shown in Figure S2 (Supporting Information), proving the limited affected area in different directions.

Using the principle of support isolation and the mechanism of wedge development of cracks in suspended membranes, a sensor array including 100 units within 1 cm^2^ (presented in Figure 1E) is successfully fabricated. In particular, for upper sensitive structures, customized substrate is designed to form stress‐concentrated structures as shown in Figure 1F, resulting in a controlled number and location of microcracks, which are used to achieve the goal of improved consistency and controllability in the fabrication of high‐density arrays.

Figure 1G presents a performance comparison of micro sensor arrays recently reported.^[^ ^17^, ^25^, ^26^, ^28^, ^32^, ^34^, ^37^, ^38^, ^42^, ^50^, ^51^, ^52^, ^53^, ^54^, ^55^ ^]^ Red dots indicate sensor arrays considering the isolation design, while blue dots represent those that are not designed to prevent crosstalk. A comparative analysis reveals that most sensor arrays do not consider the influence of crosstalk between units, while our approach emphasizes crosstalk‐free design and thoroughly considers the primary performance indicators of distributed sensing. Notably, our distributed sensors, with a unit size of just 0.5 mm × 0.5 mm, are smaller than typical millimeter‐scale sensors, showcasing the array's significant potential for high‐density perception. Despite its compact size, this array maintains a wide measurement range (up to 400 kPa) and high maximum sensitivity (0.19 kPa^−1^), rendering it comparable to larger units and well‐suited for applications in environments where space is limited, and high‐stress is anticipated.

### 2.2 Strategy for Generating Customized Cracks and Fabrication of the Multilayer Sensor Array

**Figure 2:**
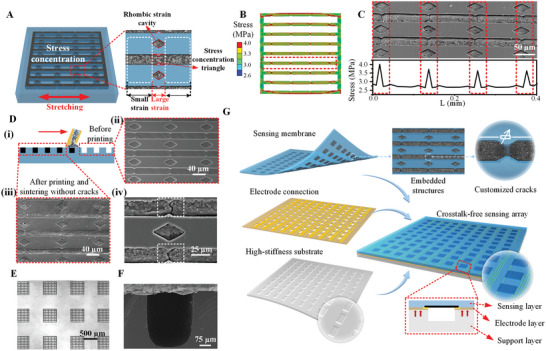
Strategy for generating customized cracks and fabrication of the multilayer sensor array. A) The mechanism of the rhombic strain cavity and stress concentration triangles to form customized cracks. B) The simulation of the CNT network in the stretching state. C) The result of stress concentration structures and customized cracks. D) (i) Micro constraint filling process to fabricate sensitive structures and SEM images before (ii) and after (iii) fabricating conductive networks. (iv) The SEM image of customized cracks induced by a set of stress concentration structures. E) The optical microscope image of the sensor array on the upper sensing layer. F) The SEM image of the cavity on the support layer. G) The multilayer structure of a crosstalk‐free sensor array including an upper sensing layer with sensing microcracks, an electrode layer, and a structured support layer.

Customizing crack structures in the suspended deformation sensing membrane is key to achieving high‐performance sensing. Although crack sensing is highly sensitive, uncontrolled crack generation leading to random microcracks can affect the stability of the sensor array. Therefore, we employ a substrate with stress concentration features to facilitate microcrack generation in the sensitive material. **Figure** **2** illustrates the specific principle of customized cracks and the structured strategy of a multilayer crosstalk‐free sensor array. The customized crack method is testified through simulation and structure analysis. Each customized crack zone is similar to a variable resistor, and the crack resistors in the same microchannel are connected in series. When the crack expands, the overall resistance of the material increases due to the fracture of the conductive path.

Figure 2A highlights the controlling mechanism of customized cracks by stress concentration structures. In the model where the conductive filler CNT network is embedded in a PDMS substrate, significant mechanical property differences are formed. PDMS has excellent ductility and flexibility, while CNTs, despite their extremely high tensile strength, have relatively low ductility and more defects. When the PDMS elastomer is stretched, the internal CNT network, due to differences in elasticity, bears more load and is more prone to fracture. The parts that are more likely to fracture often occur at stress concentration areas, that is, at sections with dangerous cross‐sections. By fully utilizing the structures that lead to dangerous cross‐sections, the formation of microcracks can be controlled. The stress concentration structures we designed are primarily composed of stress concentration triangles and rhombic strain cavities, as shown in the magnified image in Figure 2A. In the design, the purpose of using stress concentration triangles is to reduce the cross‐sectional area of the conductive material within the same channel. According to material mechanics formulas *σ* = *F*/*A*, under the same tensile force *F*, the local stress *σ* in the region with the smallest cross‐sectional area *A* is the largest, so cracks are more likely to occur here. Since the CNT channels with the stress concentration triangles have a smaller cross‐sectional area, when a tensile force is applied, the stress levels in these areas are higher. Higher stress levels mean that the mechanical load on the materials in these areas is greater, increasing the likelihood of crack formation. By precisely controlling the location and size of these stress concentration triangles, the potential crack paths in the CNT filling material can be predetermined. On the other hand, the rhombic strain cavities are more likely to contract inward under tensile stress and produce greater strain along the direction of stretch. This significant strain can be transferred to the CNT network through the friction at the contact surfaces. Consequently, the CNTs in these areas are prone to experiencing greater strain compared to other regions, leading to fractures. By integrating the stress concentration areas formed by the combination of the two aforementioned structures, it is possible to strategically guide the formation of microcracks, thereby further controlling and optimizing the macroscopic behavior of materials.

The detailed derivation of the formula concerning the combined function of the rhombic strain cavity and stress concentration triangles is shown in Figure S3 (Supporting Information), with simulation verification presented in Figure 2B and Figure S4 (Supporting Information). In the PDMS substrate, CNT microchannels of uniform size are embedded. When a 0.1% lateral deformation is applied, greater stress concentration is observed at specially designed nodes. The stress distribution curve on one of the CNT channels is intercepted as shown in Figure 2C, which is consistent with the actual microcrack distribution in the SEM image. This embedded type of customized cracks can obtain highly sensitive sensing and inhibit stray cracks by stress concentration, thus improving the stability of flexible sensors.

The fabrication of high‐precision sensor line widths is key to obtaining a flexible, high‐density array. Unlike the traditional method of printing sensitive materials on a thin‐film substrate, this paper proposes embedding printed sensitive materials into the prefabricated substrate to enhance printing accuracy and adhesion stability. The customized structure is mainly fabricated by a high‐precision printing process as shown in Figure 2D(i), which is achieved by microchannel constraint filling. When CNT solution is introduced into a PDMS substrate with microchannels, capillary forces allow the solution to flow automatically along the microchannels and fill these tiny spaces. Subsequently, under the effect of sintering, the solvent gradually evaporates, assisting in the fixation of CNTs within the PDMS. This process enables the formation of a more stable and robust structure of the conductive material within the microchannels, with increased density in the network. After sintering, there may be some unfixed or loosely attached CNTs on the surface of PDMS. When wiping the PDMS surface with a dust‐free cloth moistened with anhydrous ethanol, the weak binding force between the CNTs and PDMS surface, along with the reduction of interface contact facilitated by ethanol, allows the surface CNTs to be wiped away. The CNTs within the microchannels have already formed a relatively strong structure with PDMS during the sintering process, and therefore, they are not easily removed. This process is crucial for the manufacturing of multi‐pixel sensor arrays, ensuring their intricate design and functionality.

The SEM images of the structured substrate and the embedded sensitive network, shown in Figure 2D(ii),(iii), confirm that the substrate's network effectively constrains the sensitive material, particularly in forming the critical cross‐section, as illustrated in Figure S5A (Supporting Information). After stretching, the stress concentration structures effectively control the regions of CNT crack formation, as highlighted in Figures 2D(iv) and S5B (Supporting Information). During sensing signal transmission, the generated sensitive structures keep microcracks in the stress concentration area closed (Figure S5D, Supporting Information). These microcracks expand upon PDMS deformation due to back pressure (Figure S5E, Supporting Information), confirming the sensing mechanism. To enhance the evident controlling function of the stress concentration structures, a flat CNT film on PDMS substrate is fabricated by a spin‐coating process as shown in Figure S5E (Supporting Information). The same sintering and pre‐stretching conditions are applied and finally, the microcracks are random and uncontrollable, which negatively influences the stable and customized performance of the device. The constraint printing process, as illustrated by the optical microscope image in Figure 2E, is especially suited for manufacturing high‐density arrays. It allows the formation of isolated sensing units using a thin film array of constrained elastomers, which are suspended on a high‐stiffness substrate with cavity structures (Figure 2E) to construct a pressure sensing array.

The specific construction method of the pressure sensing array is shown in Figure 2G. The upper layer is a sensing array membrane including 100 sensor units. The middle one is the electrode layer, which is produced from PI covered with structured copper, acting as a stable electrical connection. The exposed copper wires have two contactors distributed on both sides of each sensor unit, reinforced by conductive silver paste so that the varying electrical signal can be transmitted from the sensitive layer to each conductive wire and then output to the acquisition system. The bottom layer, made of light‐cured resin, features an array of cavity structures, directly below and aligned with the center of each sensitive unit in order to provide upper units with sufficient deformation space and solid support. Figure S6 (Supporting Information) illustrates the manufacturing methods of the three layers: the upper sensing membrane is based on the high‐precision constraint printing process; the middle layer is fabricated by photolithography, etching, and laser ablation processes using PI covered with a flat copper film; and the bottom support layer, which after being UV cured, is peeled off from a convex template.

Furthermore, to provide sufficient deformation space and stable electrical connections for every sensing unit, the cavities are designed to be rectangular. The longer side provides support for the electrode, while the portion extending beyond the square sensing unit ensures a sufficient deformation margin, as shown in Figure S7A (Supporting Information). The arrangement of square cavities and the transverse opposite electrodes at both ends enhance the sensor response to resistance changes in the direction of crack expansion. As shown in the equivalent circuit in Figure S7B (Supporting Information), each expandable customized crack equivalent to a variable resistor is oriented in the same direction, making the entire unit more sensitive to lateral strain. Longitudinal strain, on the other hand, merely results in the separation of the CNT network from the PDMS substrate, ultimately enabling differential detection of strain in different directions. This multilayer structure also has good flexibility. Figure S8A (Supporting Information) clearly shows the three‐layer structure and demonstrates its good bendability. The high‐stiffness substrate can also be bent by the limit and return to its original state, as shown in Figure S8B (Supporting Information).

### 2.3 Crosstalk‐Free Performance Verification of the Sensor Array with a High‐Stiffness Substrate

**Figure 3:**
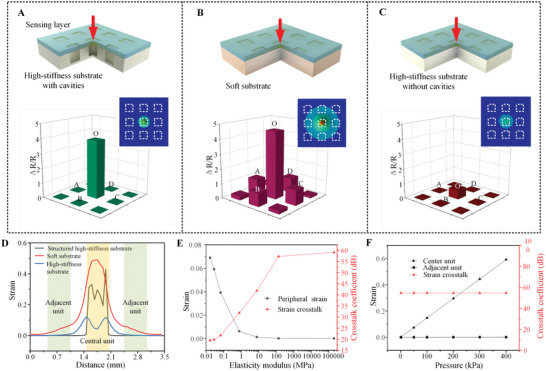
Crosstalk‐free performance verification of the sensor array with a high‐stiffness substrate. A) The simulation and the measurement of the flexible PDMS membrane on the high‐stiffness substrate with cavities. B) The simulation and the measurement of the flexible PDMS membrane on the Ecoflex flat substrate. C) The simulation and the measurement of the flexible PDMS membrane on the support substrate without cavities. D) The strain influence range curves of three different support substrates. E) The change of strain and CCR corresponding to the elastic modulus of different materials. F) The change curve of the crosstalk‐free ability of the sensor array under different pressure states.

The crosstalk‐free effect is verified with simulation and laboratory tests. To illustrate the influence of the central unit on adjacent units, a 3 × 3 array was chosen for analysis, and pressure was applied exclusively to the central unit, ensuring no interference with the others. As shown in **Figure** **3**A–C, we tested three different combinations of the proposed sensing array membrane with a high‐stiffness light‐cured resin substrate with cavities corresponding to every unit, an Ecoflex substrate without cavities, and a support substrate without cavities to highlight the unit isolation property and enhance the sensing response of our sensor arrays. In the simulation analysis and contrast experiments, the same pressure (200 kPa) was applied to the central unit (unit‐O) of the upper membrane in the different substrates mentioned above. Differences in material properties were highlighted by referencing different elastic moduli: the light‐cured resin, with its higher modulus, is less deformable under identical pressures compared to PDMS, whereas Ecoflex, having the lowest modulus, deforms the most.

When the central unit of the sensing membrane is pressed against the support substrate with cavities, as seen in Figure 3A, large strain occurs only in the pressurized unit, with no evident strain distribution in other units. This demonstrates that the central unit is effectively isolated under these conditions. After replacing the substrate with an Ecoflex substrate without cavities, the pressure's effect on the central unit extends to most areas of the sensing membrane, as observed in Figure 3B, causing obvious strain in adjacent units. When the sensing array membrane is placed on the substrate without cavities, as shown in Figure 3C, although the pressure‐affected range is limited, the strain in unit‐O is small, indicating the low sensitivity of the sensor array. In the experimental section shown in Figure 3A–C, the resistance change rate of the central unit and the other 8 adjacent units (units A to D and diagonal units in the test sample) are measured separately, supporting the same conclusion. Additionally, Figure S9 (Supporting Information) illustrates that the strain does not show a cumulative effect on the central unit of the supporting structure with cavities during the application of circulating pressure, allowing for an instant cycle measurement.

Furthermore, in order to evaluate the crosstalk‐free effect of different sensing structures mentioned above, the crosstalk coefficient of resistance change rate (CCR)^[^ ^50^ ^]^ is introduced:

(1) $$\mathrm{CCR}\;=\;20\mathrm{lo}g_{10}\left(\frac{\Delta R_{c\;}}{R_{c}\;}/\frac{\Delta R_{a}}{R_{a}}\right)$$

where Δ*R_c_*/*R_c_* denotes the resistance change rate of central unit applied pressure, and Δ*R_a_*/*R_a_* denotes the average resistance change rate of adjacent units. According to the definition, the higher the CCR value, the better the anti‐crosstalk performance of the array is. Results in Figure S10 (Supporting Information) show the CCR value of designed support substrate is 47.24 dB, much higher than those of the Ecoflex substrate (17.14 dB) and support substrate without cavity (29.99 dB), which confirms our sensing structure with appropriate material owns the best crosstalk‐free effect in the testing structures. Especially, though a flat membrane gives stable support to the upper membrane, the CCR value is undermined by the comparatively small resistance change rate of central unit leading to non‐negligible change of adjacent units.

Figure 3D captures the strain distribution of the upper sensing membrane passing through the midpoints on both sides of the central unit in the three models, which also shows the model including a stable substrate with corresponding cavities having the highest concentrated strain. It is worth noting that the larger strain‐influenced area can be verified in the support substrate without cavities compared to the first model because the pressure is applied to the whole substrate through the sensing membrane rather than only concentrated on the part with cavities providing deformation space. Besides, the elastic modulus is the key material parameter that influences on crosstalk performance, so we investigate 7 representative materials with different elastic modulus^[^ ^49^, ^56^, ^57^ ^]^ (detailed parameters are listed in Table S1, Supporting Information) to be the substrate with cavities and output curves of strain and crosstalk effective shown in Figure 3E. Likewise, crosstalk coefficients of strain is defined as:

(2) $$\mathrm{CCS}=20\mathrm{lo}g_{10}\left(\varepsilon_{c}/\varepsilon_{a}\right)$$

where *ɛ_c_*/*ɛ_a_* denotes the strain ratio of a central unit and adjacent units. With the increase of elastic modulus, the strain of the peripheral unit decreases gradually, and the CCS value experiences an evident increase, which means the anti‐crosstalk ability becomes better. Additionally, the performance of the light‐cured resin mainly used in our original multilayer structure is close to that of a fully rigid structural steel substrate, which demonstrates that this material is an excellent combination of stable support and flexibility. If the pressure on the central unit increases dramatically, the response of the adjacent unit shows a limited increase and the CCS nearly remains constant (56.44 dB) shown in Figure 3F. The aforementioned simulation curve proves that the anti‐crosstalk performance still exists when the sensor array takes a wide range of measurements.

### 2.4 Performance Control and Characterization of the Flexible Sensor Array

**Figure 4:**
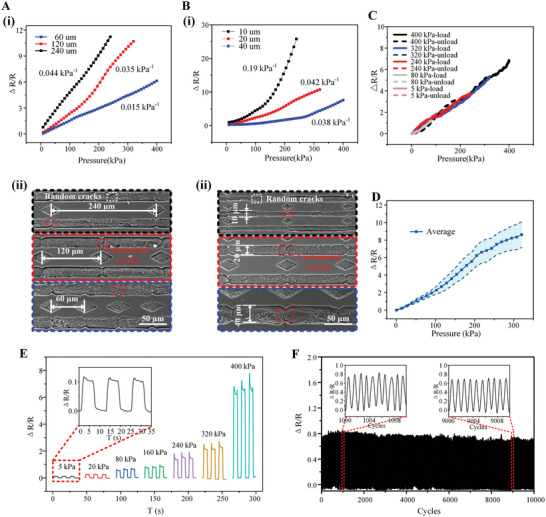
Performance control and characterization of the flexible sensor array. A) (i) The resistance change rate of sensors with different crack periods. (ii) SEM images of structures with different crack periods. B) (i) Resistance change rate of sensors with different microchannel linewidths. (ii) SEM images of structures with different microchannel line widths. C) Hysteresis characterization of the sensing unit. D) Consistency characterization of different sensing units in the sensor array. E) Typical responses of the pressure sensor within the pressure range between 5 – 400 kPa. F) The resistance change rate during 10 000 cycles of pressuring and releasing for the sensor.

In this paper, we propose a structured microcrack conductive network for sensing. An evident advantage lies in the ability to control the performance by flexibly adjusting the parameters of the conductive grid structure. **Figure** **4** illustrates the sensing performance under the influence of a suspended sensitive membrane and customized microcracks, highlighting that the crack width and density directly impact the sensor's performance.

Figure 4A(i) illustrates the resistance change rate response curve of customized crack sensors with the same linewidth (20 µm) and periods of 60, 120, and 240 µm under pressure change, respectively. To control the sum of the linewidth of the CNT network in a specific area, we set the ratio of every adjacent linewidth of CNT to PDMS as 1:2. It can be seen that the sensitivity [(Δ*R/R*_0_)/Δ*P*, where *R*_0_ refers to the initial resistance and Δ*R* refers to resistance change corresponding to the pressure change of Δ*P*] of the crack sensor with a 60 µm period is the largest, reaching 0.044 kPa^−1^ with the measurement range of 240 kPa. Meanwhile, the sensor with 240 µm period cracks presents the widest measurement range (400 kPa), but its sensitivity (0.015 kPa⁻¹) is comparatively low. According to the sensing mechanism, under the same strain, the larger the crack period in the path, the more concentrated the fracture region, the deeper the crack depth, and the easier it is to expand (Figure S11A,C, Supporting Information), thus resulting in higher sensitivity. However, the crack is much more likely to break completely, leading to a smaller measurement range. Figure 4A(ii) compares SEM images of three kinds of customized crack periods mentioned above, in which random cracks may appear when the customized crack period is large. The linewidth of cracks also has a regulating effect that should not be overlooked. We tested the curve of the resistance change rate of sensors with various crack linewidths of 10, 20, and 40 µm with the same 120 µm periodic density under pressure changes, as shown in Figure 4B(i). A 40 µm linewidth sensor has a sensitivity of ≈0.038 kPa^−1^, but its test range can reach 400 kPa, while a 10 µm line wide sensor has the highest sensitivity of 0.19 kPa^−1^ with a test range is only ≈240 kPa. According to the sensing mechanism of microcracks, the larger the width of the conductive path, the larger the strain required for complete crack fracture, leading to lower sensitivity and a larger test range. This can also be proved by the confocal microscopy measurements as shown in Figure S11B,D (Supporting Information), where the crack depth decreases with the increase of the channel width under the same strain. Figure 4B(ii) mainly compares the sensors of 10, 20, and 40 µm crack linewidths, indicating that the small linewidth (10 µm) CNT structure produces random large cracks easily, and the PDMS supporting network is easier to deform. Meanwhile, the larger linewidth network, such as 40 µm, leads to better control of cracks.

The response curves for various pressure levels show a high degree of alignment. Taking the sensor unit with a linewidth of 20 µm and a crack period of 60 µm shown in Figure 4C as an example, the microcrack structure does not propagate beyond the stable cycle. This allows it to revert to its initial state through the robust support structure within the measurement range, resulting in nearly overlapping response curves during the pressure application and recovery stages. Since the sensor array is designed for distributed detection, uniformity in response across the array is essential. We evaluate the resistance change rate of six arbitrarily chosen sensors within the array, as shown in Figure S12 (Supporting Information). To quantify the variation in responses among different sensor groups, the average curve of the resistance change rate for the six sensors against pressure changes, along with the range of the error band, is calculated and presented in Figure 4D. This analysis demonstrates the controllable performance of the sensor array. Thanks to the small deformation area and the stable support of the substrate, sensors based on customized cracks exhibit an ideal response speed and recovery speed. As shown in Figure S13 (Supporting Information), the rise response time is only 80 ms when pressure is uniformly increased to 20 kPa, and the recovery time is 95 ms at the same pressure level. This test confirms that the sensor has the ability to measure fast dynamic signals.

The sensor consistently exhibits precise responses across various pressure cycles. We utilize the sensor with the broadest measurement range, which features a 40 µm CNT network linewidth and a 120 µm periodic crack density. This sensor undergoes seven distinct pressure cycles, each involving three consecutive loading and unloading events, as depicted in Figure 4E. It is clear that the resistance change rate for different pressure cycles demonstrates repeatable responses, aligning closely with the resistance values corresponding to similar pressures in the calibration curve shown in Figure 4B. In Figure 4F, the sensor undergoes 10 000 cycles of loading and unloading at 20 kPa using a loading device. The resistance change rate shows only a minor increase during the initial 500 cycles, attributed to crack expansion. However, during subsequent cycles, the sensor maintains a nearly constant resistance change rate, indicating stabilized crack expansion and underscoring its durability and long‐term serviceability.

### 2.5 Applications of Sensor Arrays in Distributed Force Display and Medical Intubation Operation

**Figure 5:**
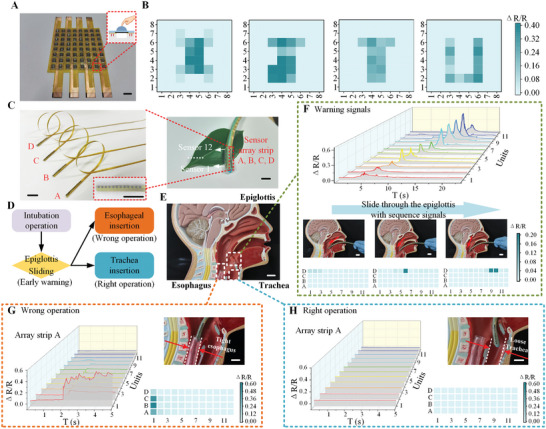
Applications of sensor arrays in distributed force display and medical intubation operation. A) The sensor array for pressure distributed display. Scale bars, 1 mm. B) Pressure cloud images of “X”, “J”, “T”, and “U” letters. C) Sensor arrays and the sensing guide wire for the intubation operation. Scale bars, 1 mm. D) The process of the intubation operation including the identification of warning signals and the comparison of right/wrong operations. E) The intubation anatomical model of the human throat. Scale bars, 1 mm. F) A sequential response of the pressure signal when the intubation guide wire slides through the epiglottis. Scale bars, 1 mm. G) The experimental scene with step response and the pressure mapping image when the intubation guide wire is wrongly inserted into the esophagus. Scale bars, 1 mm. H) The experimental scene with no evident signal and the pressure mapping image when the intubation guide wire is correctly inserted into the trachea. Scale bars, 1 mm.

In the application section, we conducted two main experiments to verify the effect of distributed force sensing and to demonstrate the essential crosstalk‐free function of the sensor array structure we proposed. In order to detect objects with flat surfaces, pressure‐sensing blocks are attached to the upper surface of each sensing pixel, as shown in **Figure** **5**A. The feature size of these hemispheres closely matches that of the sensing units. Effective bonding is achieved by curing the PDMS prepolymer that is adhered to the flat side, through contacting the upper surface of the corresponding area on the sensing membrane. The SEM images in Figure S14 (Supporting Information) present the shape of the hemispheres and prove that they can be expanded to arrays of various sizes. When a flat object presses against the entire upper surface of the sensor array, the pressure‐sensing blocks that come into contact with the object are subjected to pressure. As a result, the corresponding pixels within the sensing membrane experience strain due to the downward force exerted by the blocks. Finally, the rough outline of objects is presented with the help of a measurement system based on the principle of reference resistance voltage division, as shown in Figure S15 (Supporting Information). By pressing four letters “X”, “J”, “T”, and “U” made of metal onto the sensor array, clear pressure mapping images for each letter are shown in Figure 5B. Other pixels do not fluctuate due to the excellent crosstalk‐free performance, which is attributed to the stable support substrate designed with cavities.

In the medical application, we applied a high‐density and small‐scale sensor array to a human respiratory intubation test. Respiratory intubation is a common medical procedure with the primary purpose of ensuring that the patient's breathing tube is clear, allowing for the administration of oxygen and the maintenance of respiratory function.^[^ ^58^ ^]^ Generally, an operator opens the patient's mouth, places the laryngoscope blade correctly, and introduces the tracheal guide wire to assist in guiding the intubation tube into the trachea. Once properly positioned in the trachea, the intubation tube is secured while the guide wire is slowly removed. To detect the slow motion of the tracheal guide wire and identify abnormal intubation, four strip‐shaped sensor arrays (A, B, C, D) are uniformly adhered around the guide wire, each consisting of 12 sensor units arranged along the guide wire sequence (Figure 5C). The detection length of the sensor array strip is approximately equal to the distance from the epiglottis to the bifurcation of the trachea and esophagus, serving to monitor the process of passing the epiglottis. In the design of the sensor array, the long direction of the strips is perpendicular to the direction of crack expansion. This design makes the sensor units insensitive to the bending deformation of the strips, allowing them to only detect strain caused by pressure, thus decoupling other interference deformations.

The intubation perception and judgment process are illustrated in Figure 5D. The tracheal guide wire passing through the epiglottis, an anatomical structure used to prevent accidental inhalation of food or liquids into the trachea, plays an important role in leading. Monitoring this process can help the doctor determine when the guide wire enters the trachea. The guide wire is very close to the boundary between the esophagus and the trachea when part of its forepart slides over the epiglottis. Careful attention is required from the doctor to ensure that the guide wire does not inadvertently enter the esophagus, which could lead to an incorrect insertion.^[^ ^59^ ^]^ In addition, if too much force is applied during passing through the epiglottis, it may cause damage to this tissue.^[^ ^60^, ^61^ ^]^

Here, an anatomical model of the human throat is used as the test subject, and similar PDMS models are created in the epiglottis and trachea regions to mimic the soft touch of human tissue, as shown in Figure 5E. When the guide wire gently slides across the epiglottis, each sensor on the designed array strip that comes into contact with the human tissue is activated from the head of the guide wire to the tail, inducing strain on the upper sensor pixels and producing signals as presented in Figure 5F. Iconic moments are captured when the four sensor array strips respond, indicating the corresponding position of the guide wire. It is noticeable that the pressurized sensors within a single sensor array strip emit spike signals sequentially from one end to the other, while the other three strips remain unresponsive. This indicates that this section of the guide wire smoothly passes through the epiglottis. Benefiting from the excellent crosstalk‐free characteristic, only sensors on the contact side of the sensor array strip present typical sequential signals.

When the tracheal guide wire is wrongly inserted into the esophagus, simulated with annular PDMS film, the narrow esophageal space will squeeze the forepart of the guide wire, exerting pressure on the attached sensor^[^ ^62^ ^]^ (as shown in Figure 5G). Figure S16A–C (Supporting Information) supplements the dynamic output of three other sensor array strips in the test. Only sensors distributed around the front part that are squeezed produce noticeable step signals, while the rest respond weakly due to the crosstalk‐free design. Conversely, correct insertion into the loose trachea will not lead to changes in the sensors because there is no evident pressure, as shown in Figure 5H. Similarly, Figure S16D,E (Supporting Information) supplements the stable response and forms the pressure cloud map with no significant response. By observing the varying responses of the distributed sensors, the operator can clearly identify the incorrect and correct intubation positions. In conclusion, these three procedures compare the different distributed responses of sensor arrays, confirming that this high‐density, crosstalk‐free sensing strategy is highly effective for detection and identification in limited‐space operational environments.

## 3 Conclusion

We have fabricated a bioinspired sensor array with a prominent crosstalk‐free capability and excellent sensing performance under miniaturization, enabling high‐density integration and flexible variable‐size design. Specifically, a suspended sensing membrane is attached to the electrode layer and a high‐stiffness substrate, providing stable support and sufficient deformation space for sensing. This design ensures a balance between sensitivity and measurement range while preventing the mechanical influence of other sensing units on the measured unit, leading to crosstalk isolation of the sensing membrane. Moreover, we employ high‐precision printing and a flexible PDMS substrate with networks to fabricate embedded CNT materials. This strategy assists in forming wedge‐shaped channels, which are not easily fractured at the beginning of applying pressure, thereby improving the measuring range while retaining microcrack sensitivity.

Stress concentration structures are incorporated into the design of the sensing membrane as well. The combination of rhombic strain cavities and stress concentration triangles limits the areas of microcracks and prevents random structures. Using this controllable process, the performance of sensing units can be adjusted by the crack density and linewidth, achieving a balance between sensitivity and measuring range. Finally, a high‐density sensor array (100 units within 1 cm^2^) with a prominent CCR of 47.24 dB is fabricated, achieving a maximum sensitivity of 0.19 kPa^−1^ and a measurement range of 400 kPa. We also demonstrate stable resistance rates under 10 000 cycles and a relatively narrow error band for different pixels on a sensing array. Based on the outstanding crosstalk‐free capability and excellent detection performance, we successfully realize a distributed application, and verify the characteristic response of the sensor array attached to a tracheal guide wire in an intubation test, showcasing promising adaptation in the medical field.

## 4 Experimental Section

### Fabrication of the Upper Sensing Membrane

The upper sensing membrane was based on a flexible PDMS substrate with a mutually isolated sensor structure, imprinted from a silicon template, which was fabricated by photolithography and etching. To ensure the flexible substrate can be peeled off from the silicon template successfully, a layer of C_4_F_8_, an effective hydrophobic layer was deposited on the structure surface. Then, the PDMS base (SYLGARD 184, Dow Corning) was stirred with the cross‐liner at a mass ratio of 10:1 for 10 min and degassed in a vacuum for 10 min to remove bubbles. Then, the prepolymer was spin‐coated on the template and degassed again for 10 min in order to penetrate in the microstructure area. After the curation for 2 h at 80 °C and removal from the silicon templet, the PDMS mold was well prepared. In order to prepare the embedded structure of the CNT and PDMS membrane, the prepared PDMS membrane was treated with oxygen plasma to increase the hydrophilicity so that the CNT solution could be compatible with the substrate. An aqueous solution of CNT (XFNANO, Nanjing) with a mass fraction of 10% was printed on the surface of the structural area of the PDMS. Capillary force and a hydrophilic layer enabled the CNT to be filled into the designed flow path to form a line with predesigned stress concentration structures. After sintering at 80 °C for 5 min, a dust‐free cloth containing anhydrous ethanol was used to gently wipe off the residual CNT on the surface of the PDMS. To activate and produce pre‐fabricate cracks, the embedded distributed structure of the CNT and PDMS membrane was pre‐stretched by 10%, and finally, the distributed sensing structure was prepared.

### Fabrication of the Middle Electrode Layer and Bottom Supporting Layer with Cavities

The middle electrode layer was originated from PI film covered with copper. After the photolithography process, the film was etched in ferric chloride solution with a mass fraction of 30%, thereby forming a patterned copper electrode. The PI material in the middle of the electrodes on both sides of each unit is then removed by laser etching to provide a channel for the downward deformation of the upper membrane.

The bottom support layer with cavities, being difficult to deform, was easily broken when peeled off from a normal rigid template. Thus, the PDMS prepolymer was poured onto the patterned aluminum alloy template fabricated by metal precision machining, degassed for 10 min, and then cured for 5 h at 80 °C. Next, the NOA65 (Norland) as the substrate and UV curable material was drop‐coated onto the printed PDMS template and exposed to UV light for two min. After curing, the NOA substrate was easily peeled off from the PDMS template, and then the distributed array structure was prepared.

### Fabrication of the Distributed Sensor Array

In order to enhance the adhesive force and conductivity between the upper sensing layer and the electrode layer, the conductive silver paste (Jurong Electronic Technology Co., Ltd, Shanghai) was coated on the copper contacts of the electrode layer, which was used to contact the upper sensing unit, by screen printing techniques (screen, 300 mesh). After the two layers are aligned and fitted together, they are sintered at 80 °C for 10 min to form a stable electrical connection. The bottom support layer with cavities was dropped with silica gel binder (Cutorin4672, Trane Solid Plastic Co., Shenzhen) and also was adhered to the lower side of the middle electrode layer in a right position corresponding to the upper units. Once the silica gel binder was dried up at room temperature, the multilayer sensor array was well prepared.

### Characterization

The morphologies of the structured substrate and microcracks embedded in the microchannels were all characterized by SEM (SU8010, HITACHI). The dimensional characteristics of microcracks were obtained with a laser scanning confocal microscope (OLS4000, Olympus). In order to generate uniform microcracks, the sensing membrane was stretched using a ball screw with a stepper motor, a controller, and a fixture design. For the pressure test, a custom indenter applied pressure through a tensile machine (ESM303, Mark‐10), and the resistance change of each sensor was measured through a source meter (B2912A, Keysight). The curve of the pressure and resistance change with respect to time can be recorded by the instrument, and the resistance‐pressure curve can be obtained by replacing the instances corresponding to different resistances with the respective pressure. Crosstalk testing and pressure distribution display applications were conducted using a combination of commercial data acquisition cards (ART) and a built PCB. Shunt voltages were collected and compared with voltages of fixed resistors in series to determine the resistance of each sensor.

### Statistical analysis

In the characterization of sensor performance and microcrack morphology, tests that need to prove universality involve more than five samples, as specified in the corresponding sections of the document. For performance characterization, small sensor units measuring 0.5 mm × 0.5 mm are used for standardized testing.

## Conflict of Interest

The authors declare no conflict of interest.

## Author Contributions

H.L., X.C., and J.S. conceived the concept; H.L., J.X., and M.Z. carried out experiments and conducted the simulation. H.L., X.L., H.T., C.W., and B.S. analyzed and integrated the data; H.L., J.H., and B.L. analyzed and practiced the feasibility of devices for medical applications; H.L. and S.L. contributed to the construction of the measurement system; H.L. and X.C. wrote the paper. All authors reviewed the manuscript.

## Supporting information

Supporting Information

## Data Availability

The data that support the findings of this study are available on request from the corresponding author. The data are not publicly available due to privacy or ethical restrictions.
