# Supplementary material for: Bioinspired Suspended Sensing Membrane Array with Modulable Wedged‐Conductive Channels for Crosstalk‐Free and High‐Resolution Detection
Source: Adv Sci (Weinh). 2024 May 8;11(28):2403645. doi: 10.1002/advs.202403645 (PMC11267273; doi:10.1002/advs.202403645)
Supplement: Supplementary file 1 — Supporting Information [file ADVS-11-2403645-s001.docx]

Supporting Information

Bioinspired suspended sensing membrane array with modulable wedged-conductive channels for crosstalk-free and high-resolution detection

Haixuan Luo, Xiaoliang Chen^*^, Sheng Li, Jinbin Xu, Xiangming Li, Hongmiao Tian, Chunhui Wang, Bo Li, Manman Zhang, Bai Sun, Juan He, Jinyou Shao^*^

Correspondence to: [xiaoliangchen@mail.xjtu.edu.cn](mailto:xiaoliangchen@mail.xjtu.edu.cn); [jyshao@mail.xjtu.edu.cn](mailto:jyshao@mail.xjtu.edu.cn)

**This PDF file includes:**

Figures S1 to S16

Table S1

Supplemental reference list

**
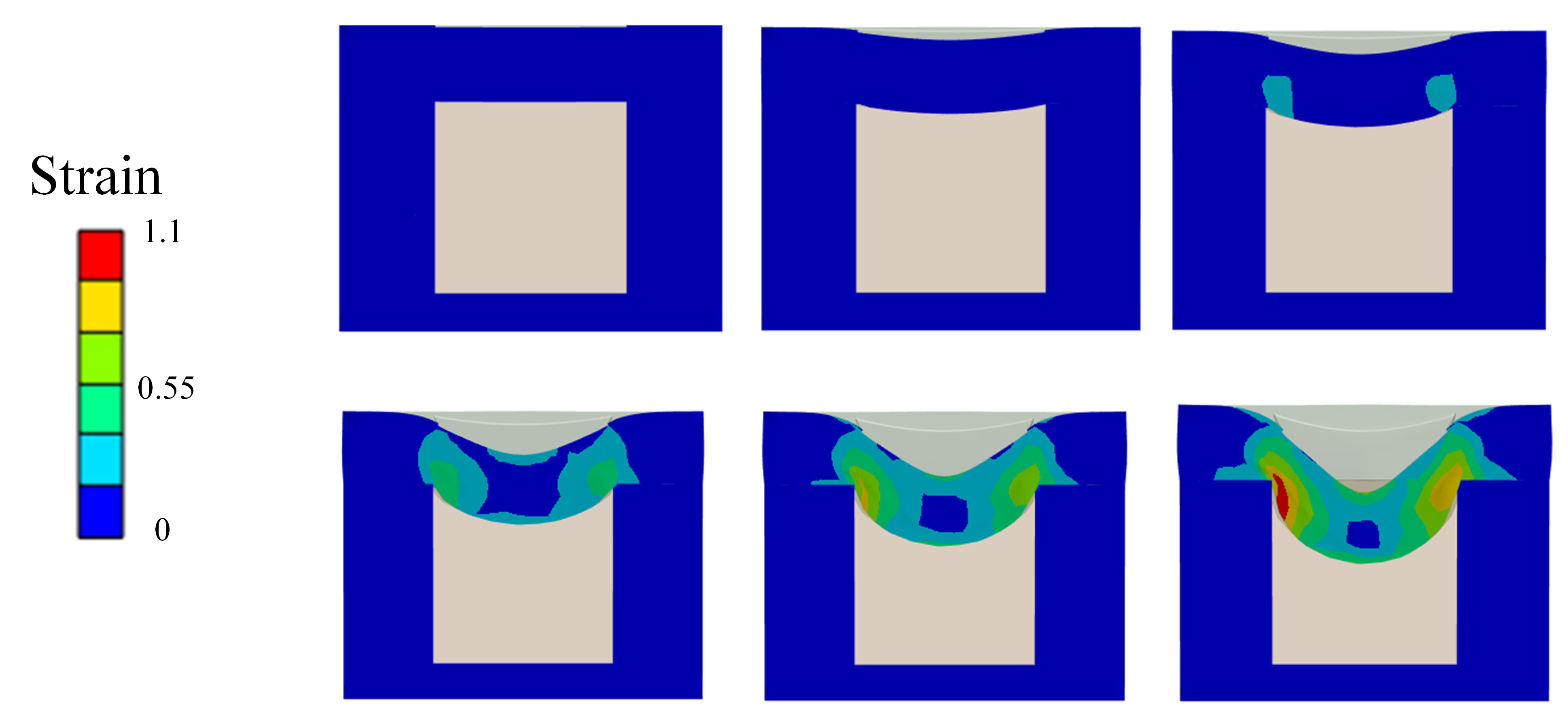
**

**Figure S1.** **Strain simulation of a single sensing unit under different pressures.** When different pressures of 0 kPa, 20 kPa, 80 kPa, 160 kPa, 240 kPa, 320 kPa and 400 kPa are successively applied to the sensor, the strain of the upper sensing membrane gradually increases, while the bottom high-stiffness support layer does not show obvious strain.


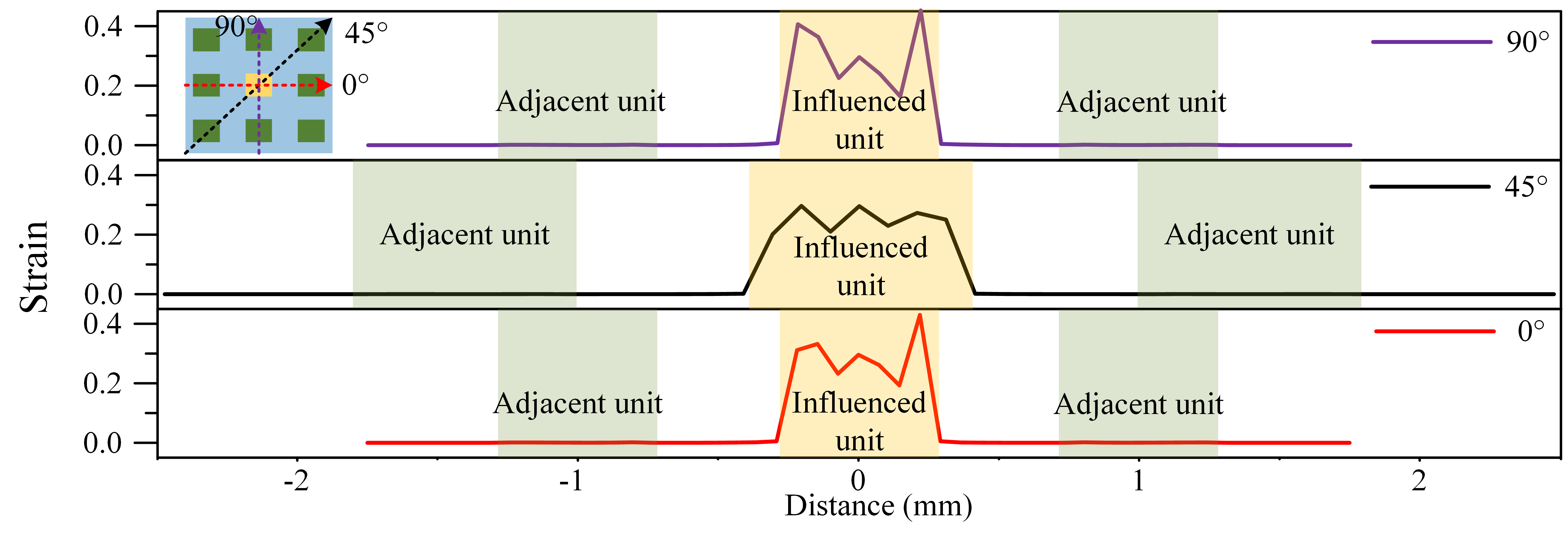


**Figure S2. Strain curves from different directions in a 3×3 flexible sensor array.** When a pressure of 200 kPa is applied, the horizontal, oblique and vertical (0°, 45°, 90°) strain distributions centered on the compression sensing unit are simulated, verifying the good support isolation effect of the high-stiffness substrate.


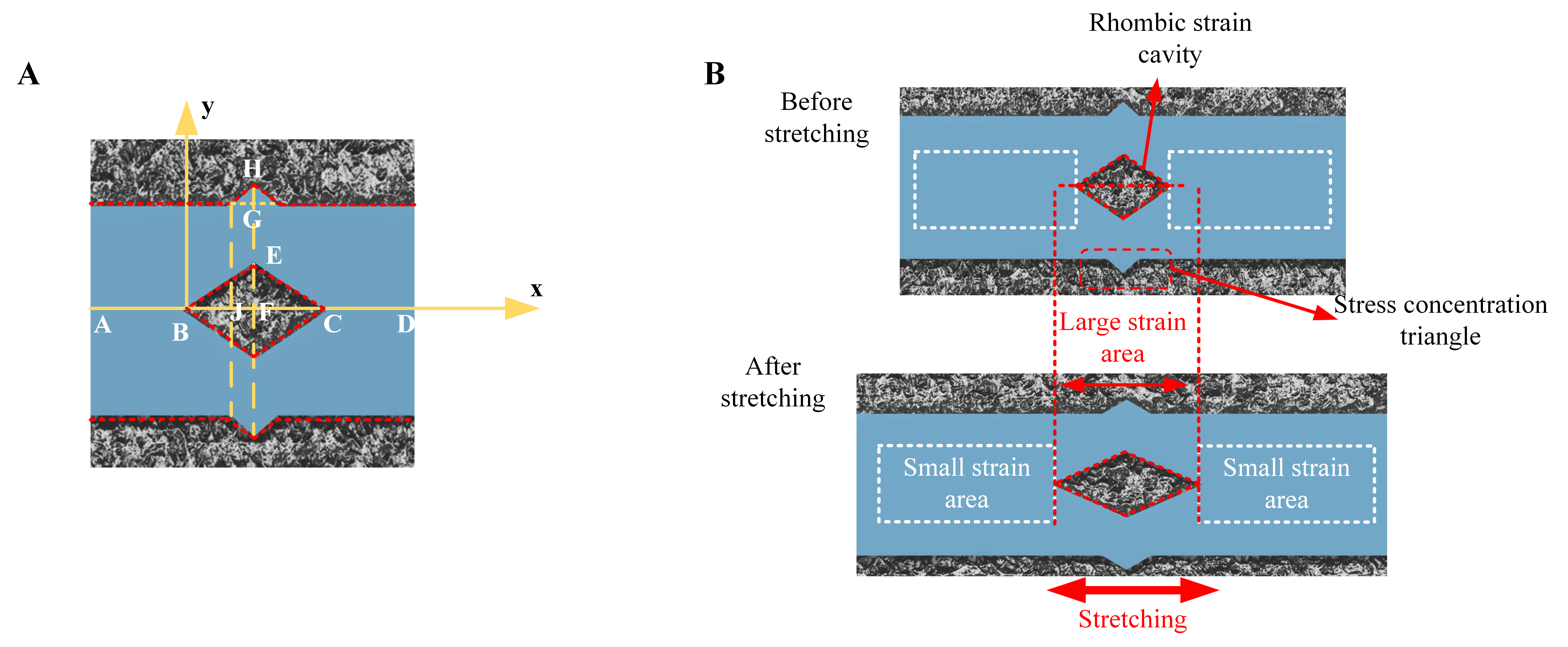


**Figure S3.** **Function mechanism of the stress concentration structure.** (A) The coordinate system of the rhombic strain cavity with stress concentration triangles. (B) The comparison of a stress concentration structure before and after stretching.

When a tension *P* is applied to both ends of the selected structure, the effects of the rhombic strain cavity and stress concentration triangles are derived as follows:

Let *AB = BC = CD = L*, *BF = FC = L/*2, *FE = a*, *EH = b* and *GH = c*.

The stress of segment *AB*:

|  | $\text{σ}_{\text{AB}}\text{ }\text{=}\text{ }\frac{\text{P}}{\text{A}}\text{ }\text{=}\text{ }\frac{\text{P}}{\text{2}\left( \text{a}\text{ }\text{+}\text{ }\text{b} \right)}$ | (S1) |
| --- | --- | --- |

The strain of segment *AB*:

|  | $\text{ε}_{\text{AB}}\text{ }\text{=}\text{ }\frac{\text{σ}_{\text{AB}}}{\text{E}}\text{ }\text{=}\text{ }\frac{\text{P}}{\text{2}\text{E}\left( \text{a+b} \right)}$ | (S2) |
| --- | --- | --- |

The deformation of segment *AB*:

|  | $\text{δ}_{\text{AB}}\text{ }\text{=}\text{ }\text{ε}_{\text{AB}}\text{⋅}\text{L}_{\text{AB}}\text{ }\text{=}\text{ }\frac{\text{PL}}{\text{2}\text{E}\left( \text{a+b} \right)}$ | (S3) |
| --- | --- | --- |

It can be seen from the symmetry that the change in displacement of segment *CD* is equal to the change in displacement of segment *AB*.

The sectional area of segment *BF*:

|  | $\text{A}_{\text{BJ}}\text{ }\text{=}\text{ }\text{2}\left( \text{a}\text{ }\text{+}\text{ }\text{b} \right) \text{-}\text{ }\text{2}\text{⋅}\frac{\text{x}}{\text{L/}\text{2}}\text{⋅}\text{a}\text{ }\text{=}\text{ }\text{2}\left( \text{a}\text{ }\text{+}\text{ }\text{b} \right) \text{-}\text{ }\frac{\text{4}\text{xa}}{\text{L}}$ | (S4) |
| --- | --- | --- |
|  | $\text{A}_{\text{JF}}\text{ }\text{=}\text{ }\text{2}\left( \text{a}\text{ }\text{+}\text{ }\text{b} \right)\text{-}\text{2}\left( \text{a}\text{ }\text{-}\text{ }\text{c} \right)\text{ }\text{=}\text{ }\text{2}\left( \text{b}\text{ }\text{+}\text{ }\text{c} \right)$ | (S5) |

The stress of segment *BF*:

|  | $\text{σ}_{\text{BJ}}\text{ }\text{=}\text{ }\frac{\text{P}}{\text{A}_{\text{BJ}}}\text{ }\text{=}\text{ }\frac{\text{P}}{\text{2}\left( \text{a}\text{ }\text{+}\text{ }\text{b} \right) \text{-}\text{ }\frac{\text{4}\text{xa}}{\text{L}}}$ | (S6) |
| --- | --- | --- |
|  | $\text{σ}_{\text{JF}}\text{ }\text{=}\text{ }\frac{\text{P}}{\text{A}_{\text{JF}}}\text{ }\text{=}\text{ }\frac{\text{P}}{\text{2}\left( \text{b}\text{ }\text{+}\text{ }\text{c} \right)}$ | (S7) |

The strain of segment *BF*:

|  | $\text{ε}_{\text{BJ}}\text{ }\text{=}\text{ }\frac{\text{σ}_{\text{BJ}}}{\text{E}}\text{ }\text{=}\text{ }\frac{\text{P}}{\text{2}\text{E}\left[ \left( \text{a}\text{ }\text{+}\text{ }\text{b} \right) \text{-}\text{ }\frac{\text{2}\text{xa}}{\text{L}} \right]}$ | (S8) |
| --- | --- | --- |
|  | $\text{ε}_{\text{JF}}\text{ }\text{=}\text{ }\frac{\text{σ}_{\text{JF}}}{\text{E}}\text{ }\text{=}\text{ }\frac{\text{P}}{\text{2}\text{E}\left( \text{b}\text{ }\text{+}\text{ }\text{c} \right)}$ | (S9) |

The deformation of segment *BF*:

|  | $\text{δ}_{\text{BC}}\text{ }\text{=}\text{ }\text{2}\text{δ}_{\text{BF}}\text{ }\text{=}\text{ }\text{2}\int_{\text{0}}^{\frac{\text{L}}{\text{2}}\left( \text{1}\text{ }\text{-}\text{ }\frac{\text{c}}{\text{a}} \right)} \text{ε}_{\text{BJ}}\text{ }\text{dx}\text{ }\text{+}\text{ }\text{2}\int_{\frac{\text{L}}{\text{2}}\left( \text{1}\text{ }\text{-}\text{ }\frac{\text{c}}{\text{a}} \right)}^{\frac{\text{L}}{\text{2}}} \text{ε}_{\text{JF}}\text{ }\text{dx}$  $\text{=2}\int_{\text{0}}^{\frac{\text{L}}{\text{2}}\left( \text{1}\text{ }\text{-}\text{ }\frac{\text{c}}{\text{a}} \right)} \frac{\text{P}}{\text{2}\text{E}\left[ \left( \text{a}\text{ }\text{+}\text{ }\text{b} \right) \text{-}\text{ }\frac{\text{2}\text{xa}}{\text{L}} \right]}\text{ }\text{dx}\text{ }\text{+}\text{ }\text{2}\int_{\frac{\text{L}}{\text{2}}\left( \text{1}\text{ }\text{-}\text{ }\frac{\text{c}}{\text{a}} \right)}^{\frac{\text{L}}{\text{2}}} \frac{\text{P}}{\text{2}\text{E}\left( \text{b}\text{ }\text{+}\text{ }\text{c} \right)}\text{ }\text{dx}$  $\text{=}\frac{\text{PL}}{\text{2}\text{E}}\text{⋅}\left[ \frac{\ln\left( \frac{\text{a}\text{ }\text{+}\text{ }\text{b}}{\text{b}\text{ }\text{+}\text{ }\text{c}} \right)}{\text{a}}\text{ }\text{+}\text{ }\frac{\text{c}}{\text{a}\text{(}\text{b}\text{ }\text{+}\text{ }\text{c}\text{)}} \right]\text{ }\text{>}\text{ }\frac{\text{PL}}{\text{2}\text{E}\left( \text{a}\text{ }\text{+}\text{ }\text{b} \right)}$ | (S10) |
| --- | --- | --- |

By designing, *c* < *a*, the deformation of *BC* is larger than *AB* and *CD*. Thus, the strain concentration structure plays a significant role in crack customization.


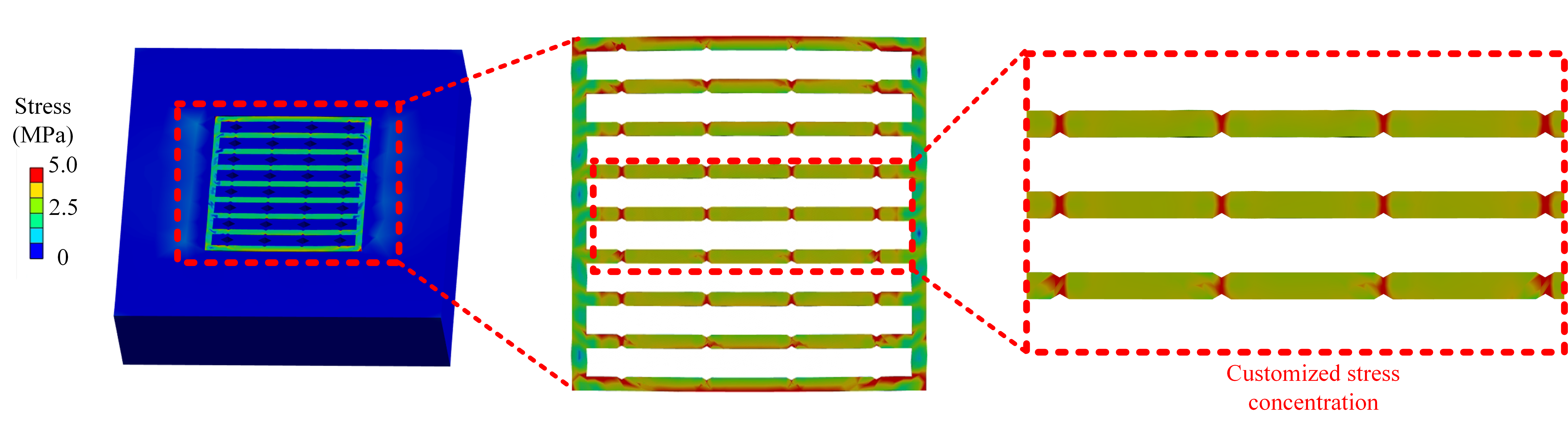


**Figure S4. Stress simulation of the CNT network embedded in the stretched PDMS substrate.** A sensing unit on the membrane is represented by embedding a sensitive material network in microchannels of the elastomer. In the simulation, the 0.1% transverse deformation is applied to the elastomer and transferred to the conductive material through interfacial friction. Finally, the conductive network shows significant stress concentration in the customized crack region, which proves the effectiveness of rhombic strain cavities and stress concentration triangles.


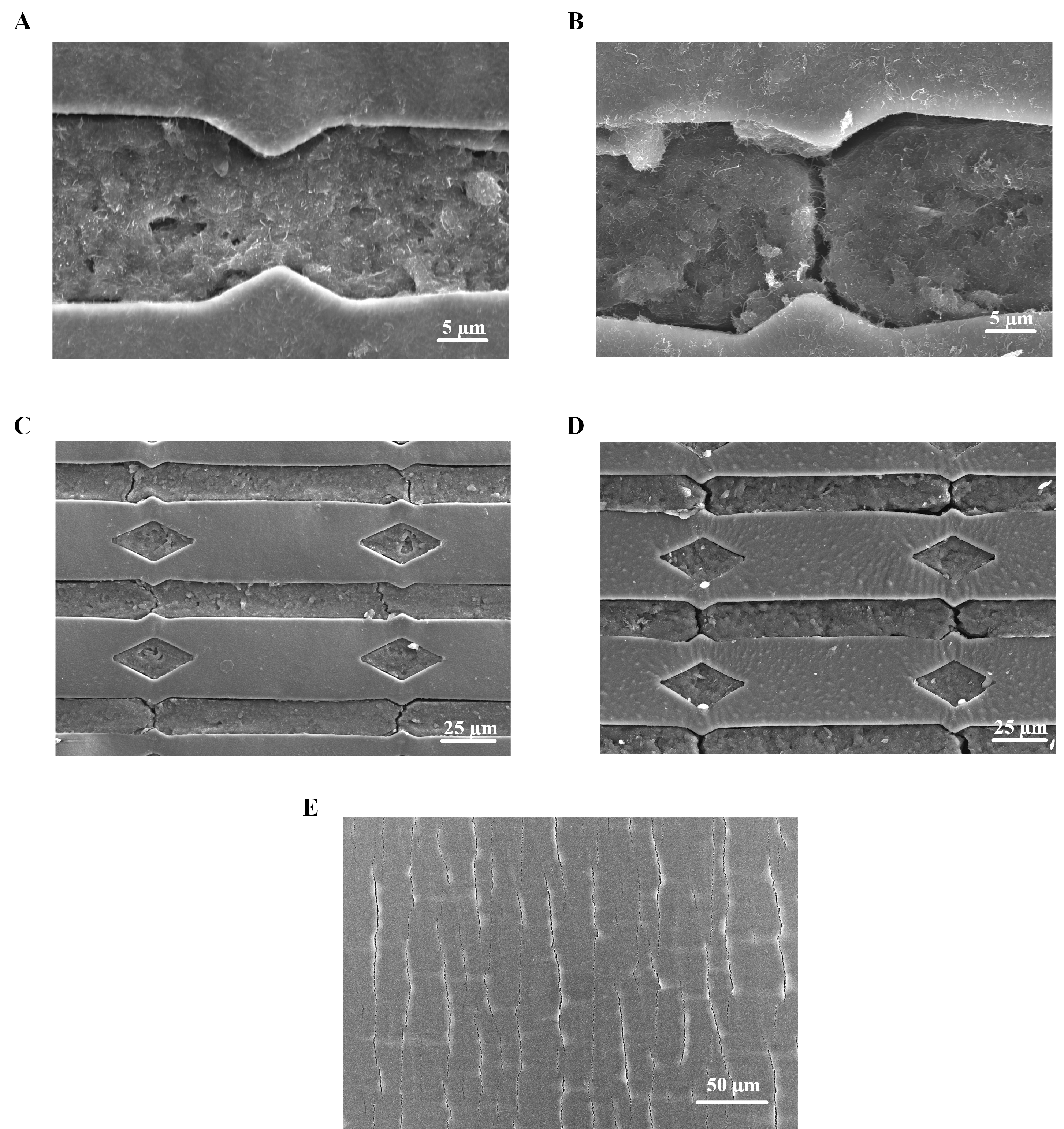


**Figure S5.** **SEM images of the** **microcrack manufacturing and development.** (A) The embedded conductive CNT structure fabricated by micro constraint printing. (B) The customized microcrack induced by stretching. (C) The non-pressure state of microcrack region. (D) The pressurized development of microcrack region. (E) The generation of random microcracks on flat PDMS flat membrane.


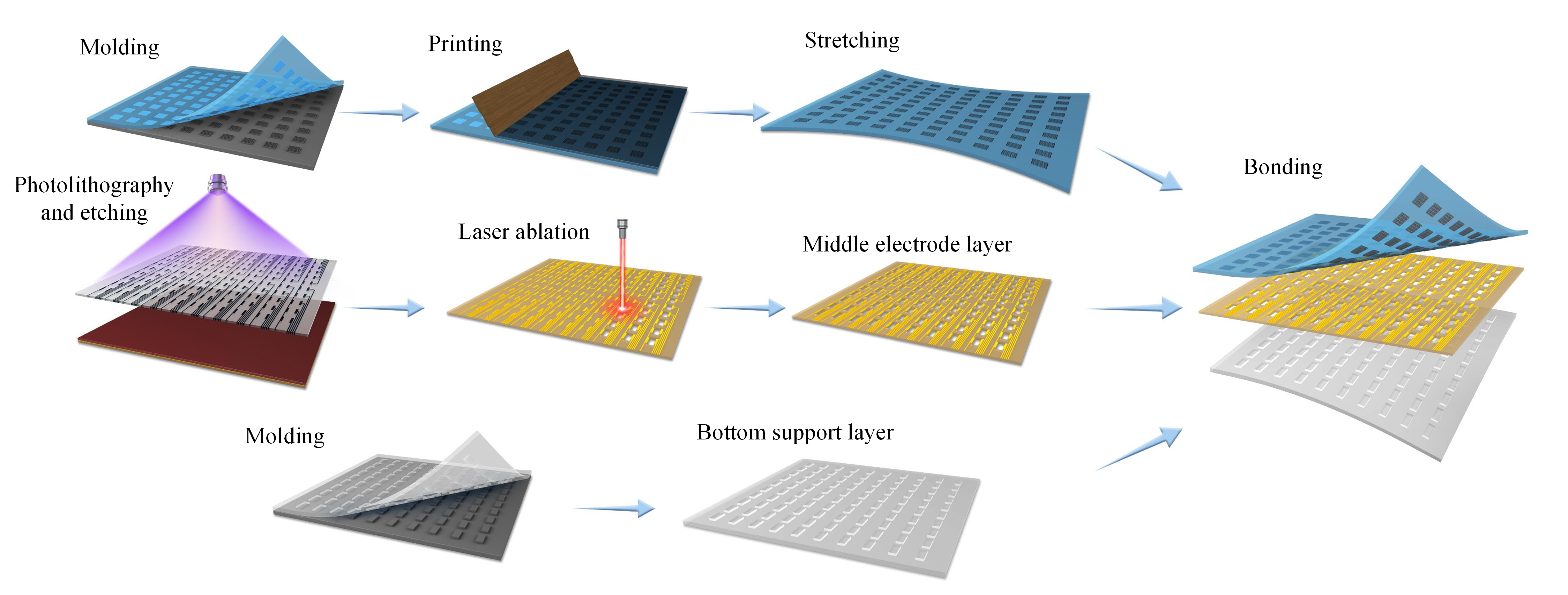


**Figure S6.** **Manufacturing process of the multilayer sensor array including an upper sensing layer, a middle electrode layer and a bottom support layer.** The upper substrate, featuring microchannels and stress concentration structures, is peeled from the silicon template. Conductive networks and sensing microcracks are then obtained through high-precision printing and a pre-stretching process, respectively. The middle patterned electrodes are fabricated using photolithography and etching, while the hollow structure at the center of each unit, designed to accommodate the deformation of the upper membrane, is created by laser ablation. The high-stiffness bottom support layer is formed through a molding process and is cured with UV light. During the bonding process, electrical connectivity is enhanced by the screen printing of silver paste, and structural support is fixed by a silica gel binder.


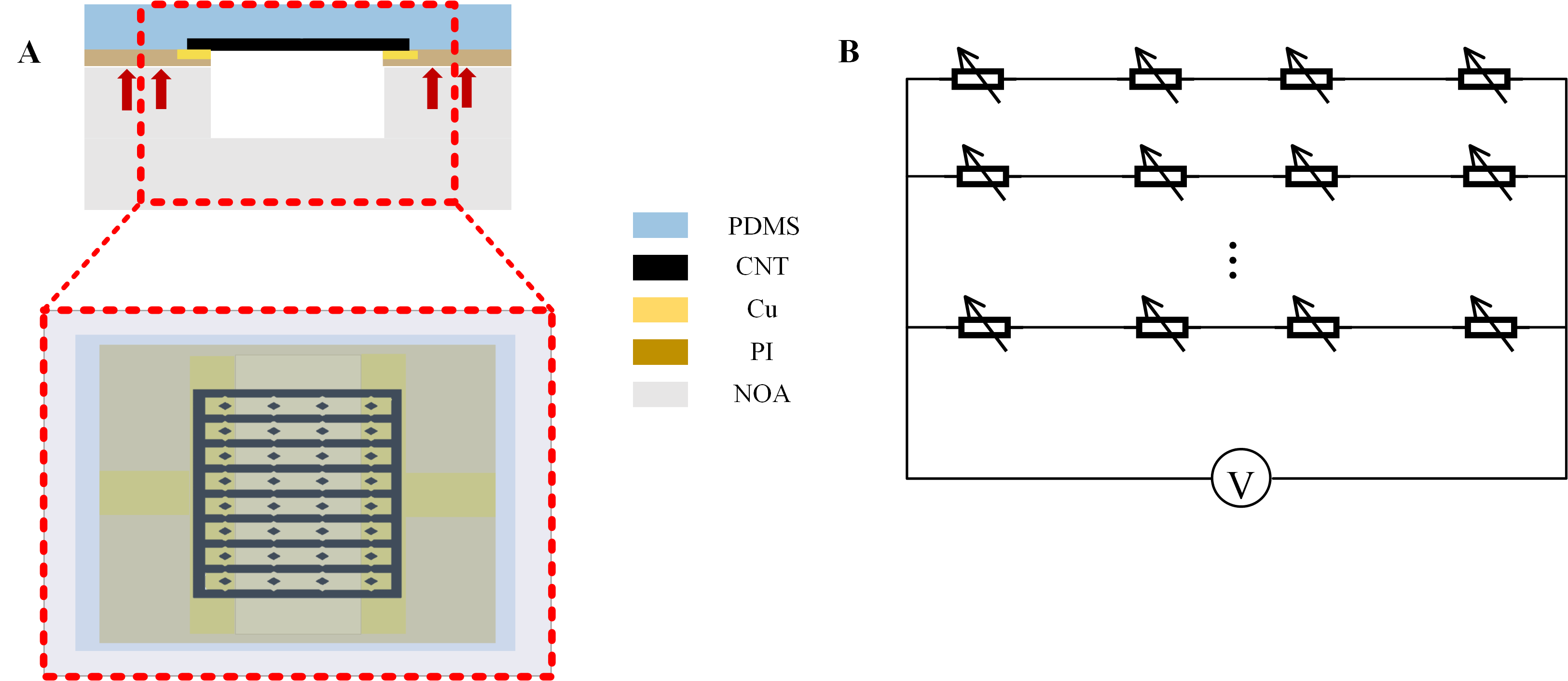


**Figure S7.** **The multilayer structure design of the sensor array and its equivalent circuit.** (A) Schematic diagram of the relative positions of different layers of the sensor array. (B) The equivalent circuit within the sensing unit.


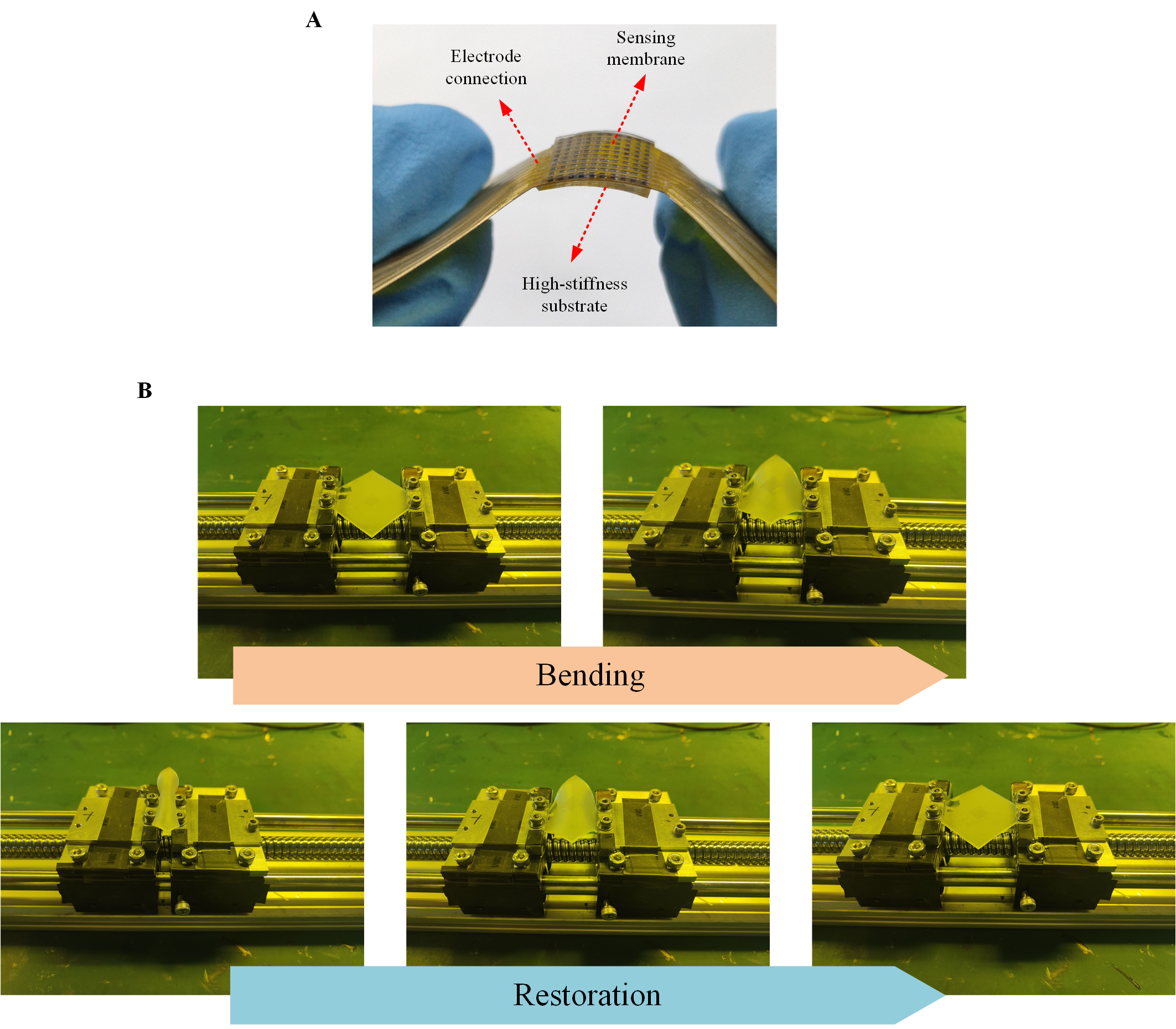


**Figure S8. Flexibility testing of the sensor array and the high-stiffness substrate.** (A) The high-density sensor array sample and flexible display. (B) The high stiffness substrate is bent on the ball screw and then restored.


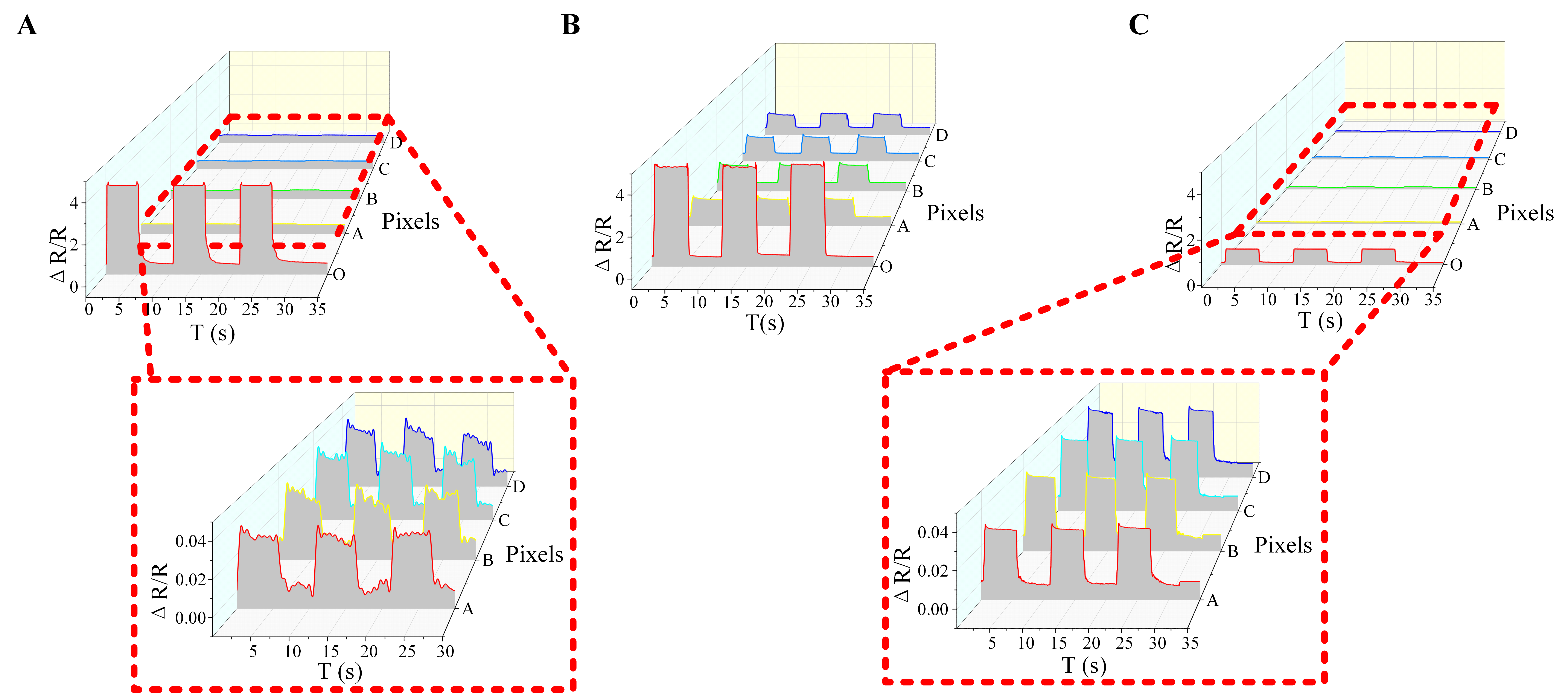


**Figure S9.** **Dynamic circulating tests under different substrate support.** The resistance change rate is tested under the action of dynamic circulating pressure of 200 kPa on the high-stiffness substrate with cavities (A), the soft flat substrate (B) and the high-stiffness substrate without cavities (C), respectively. The choice of high-stiffness material can effectively prevent signal crosstalk, and the design of structured substrate can improve the response of the sensing unit.


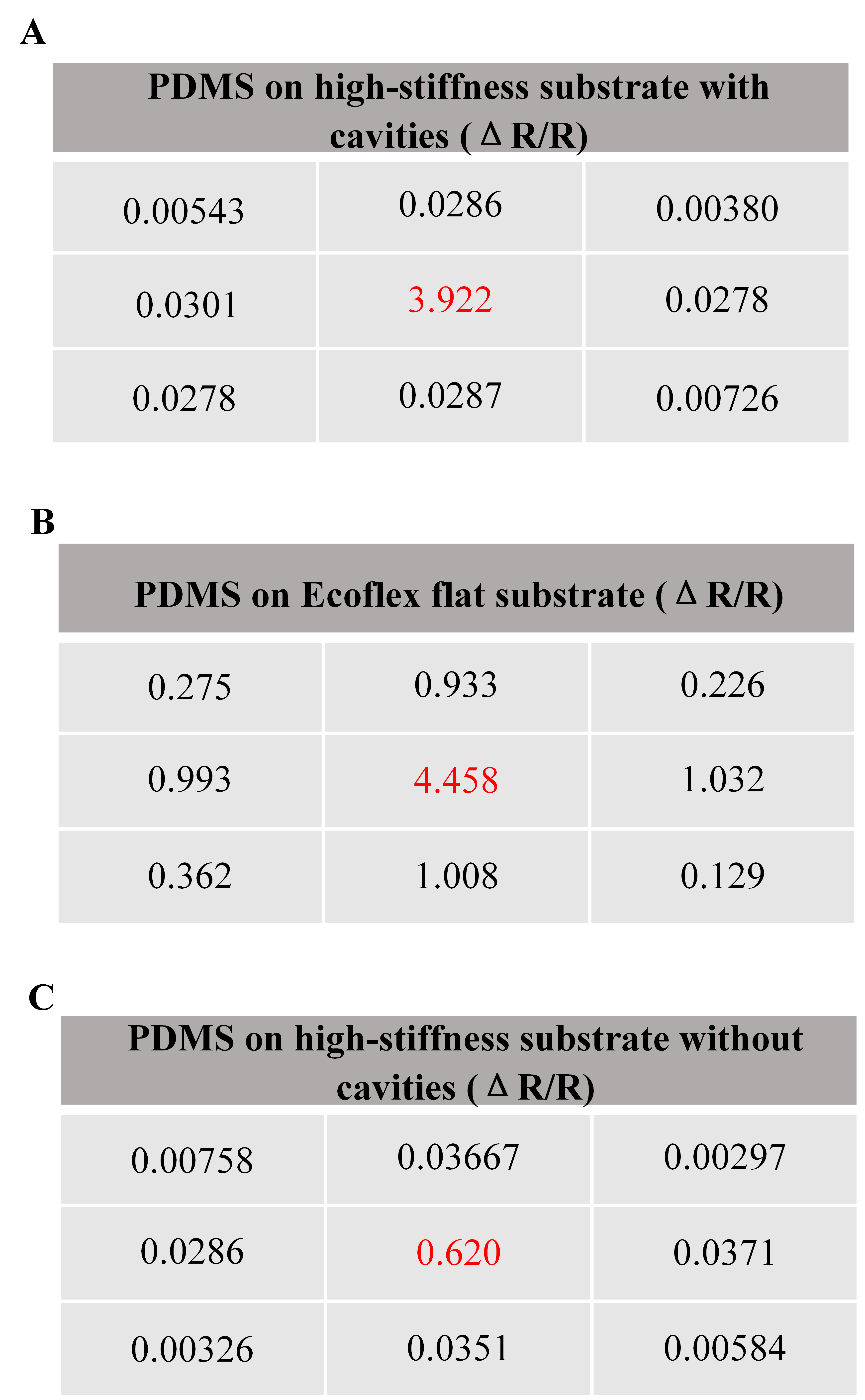


**Figure S10.** **The resistance change rate of each unit in the three typical sensor arrays under the same pressure of 200 kPa.** (A) The response of the sensor array based on the high-stiffness substrate with cavities. (B) The response of the sensor array based on the soft flat substrate. (C) The response of the sensor array based on the high-stiffness substrate without structures.


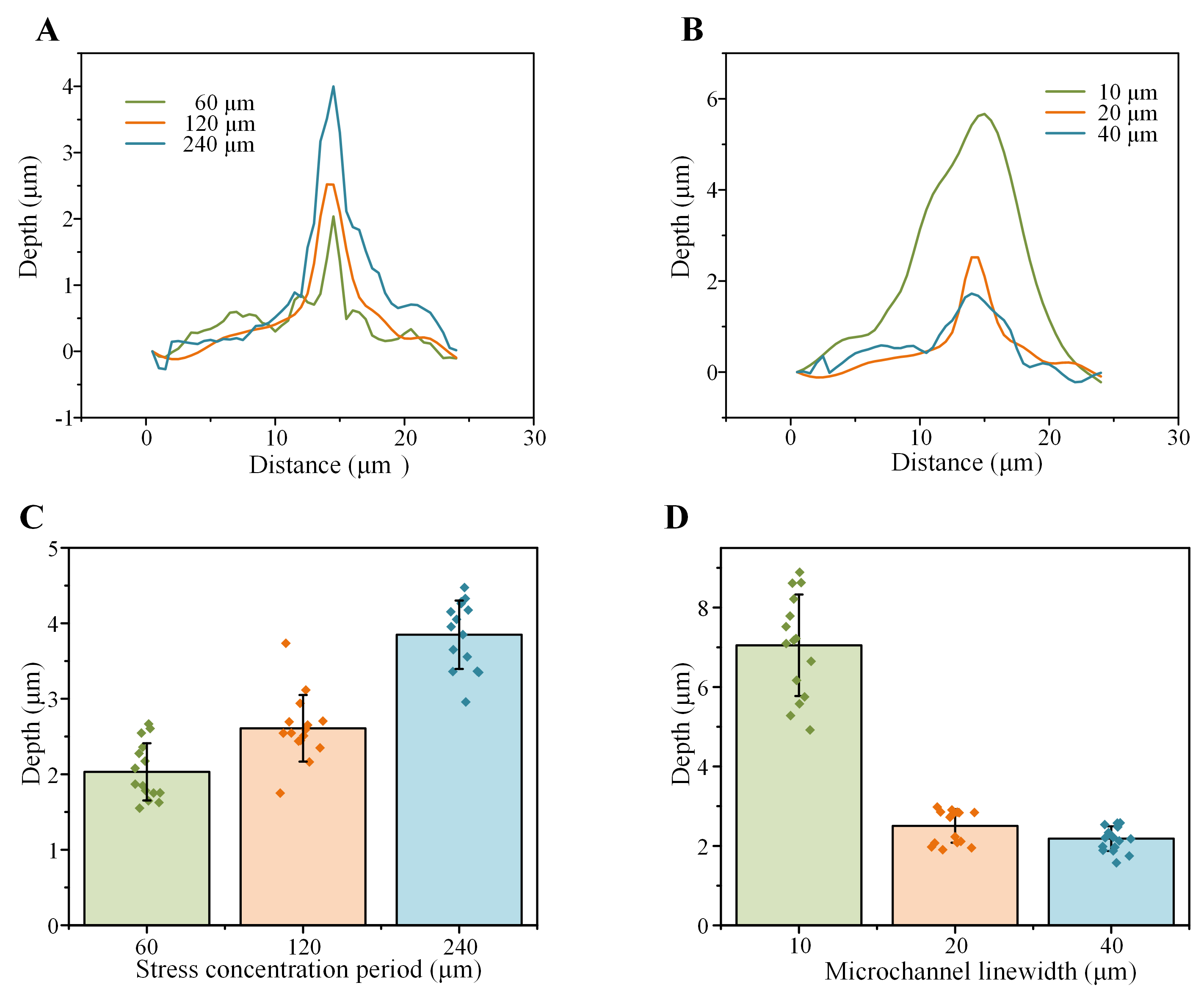


**Figure S11.** **Microcrack size characterization under different structural parameters of the substrate.** (A) Comparisons of crack depths under different stress concentration periods after recovery from the same tensile ratio. (B) Comparisons of crack depths under different microchannel linewidths after recovery from the same tensile ratio. (C) Multiple crack statistics with different stress concentration periods. (D) Multiple crack statistics with different microchannel linewidths.


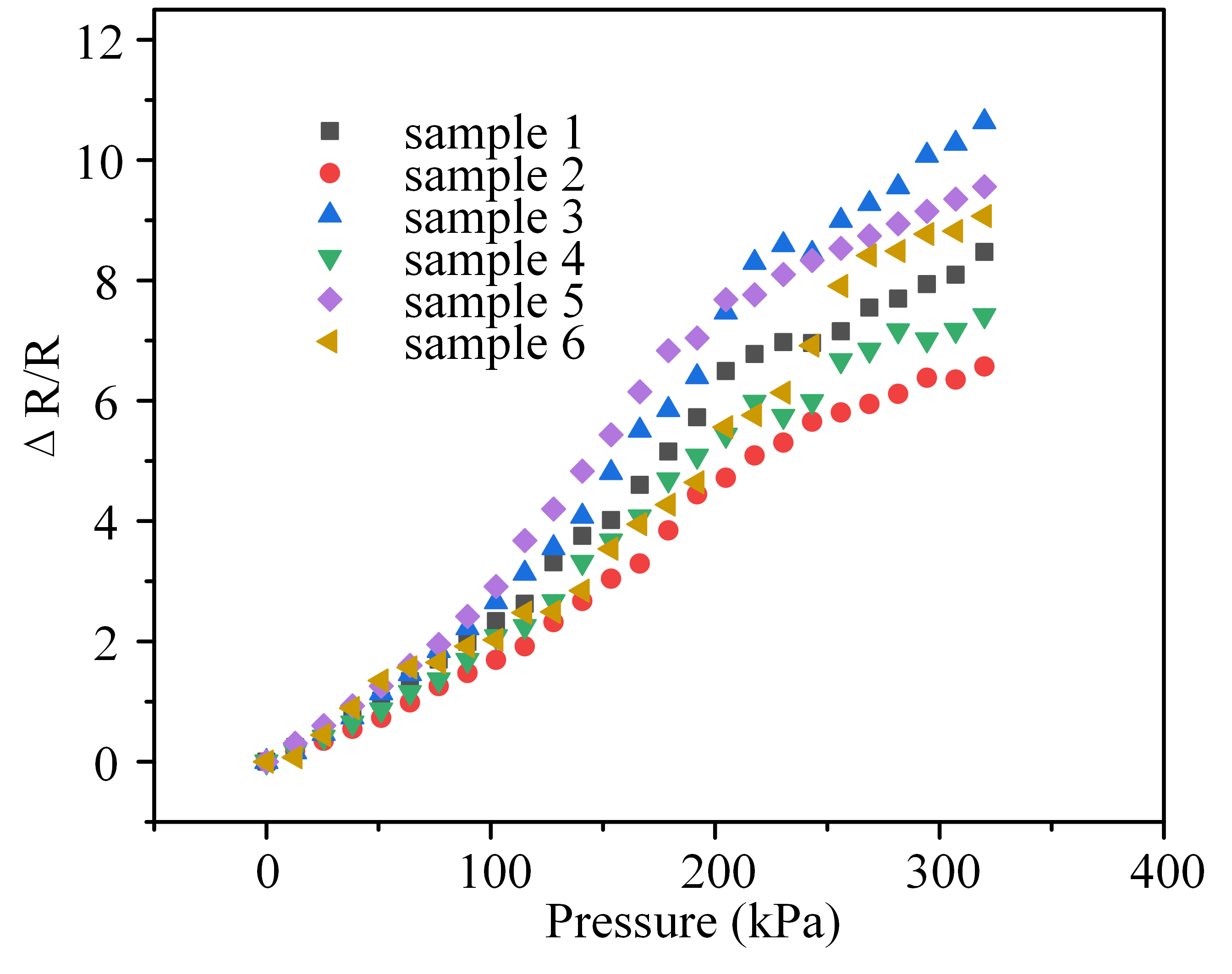


**Figure S12.** **Pressure response curves of 6 random sensors in a sensor array.** By consistent characterization of different array units, the dispersion coefficient defined as $\text{c}\text{ }\text{=}\text{ }\text{σ}_{\text{S}}\text{ }\text{/}\text{ }\overline{\text{S}}$ in statistics is used to characterize the dispersion of the array. Here, $\overline{\text{S}}$ represents the mean of multi-sensor sensitivity coefficients and $\sigma_{S}$ represents the standard deviation. The sensitivity dispersion coefficient of this sensor array based on customized cracks is 16.6%, which demonstrates a manageable range.


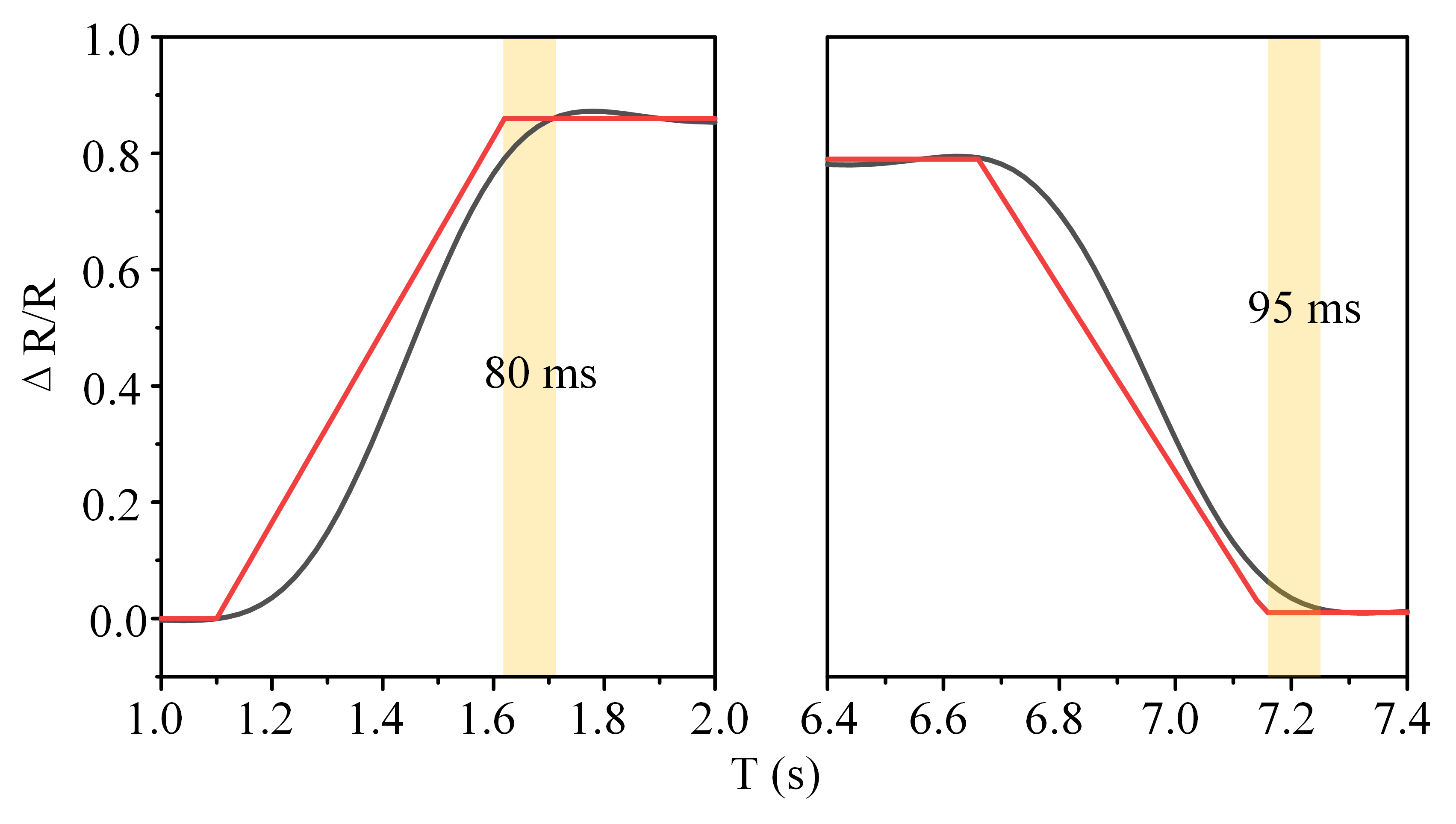


**Figure S13.** **Measurement of the sensor response time.** In this test, the rise response time is induced according to the difference between the loading time and the initial arrival time of the sensor signal, and the recovery response time is induced by the difference between the unloading time and the time when the sensor signal returns to stability.


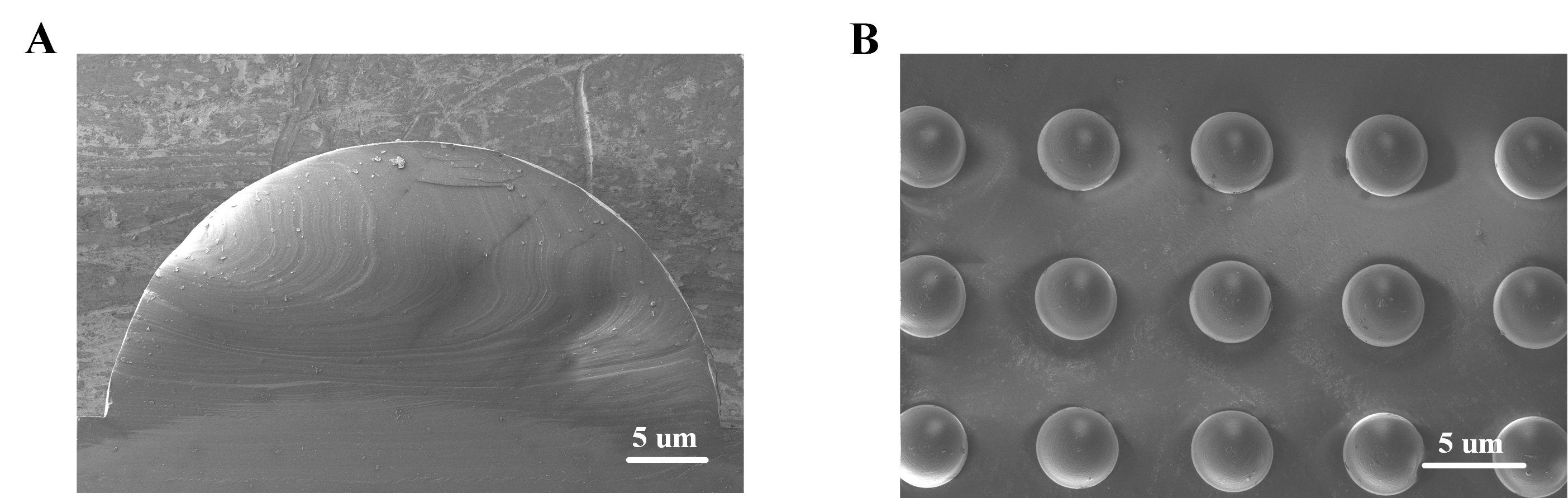


**Figure S14. SEM images of sensing blocks.** (A) A cross-section of an sensing block for displaying pressure from a rigid flat object. (B) The sensing block array for matching micro sensor arrays.


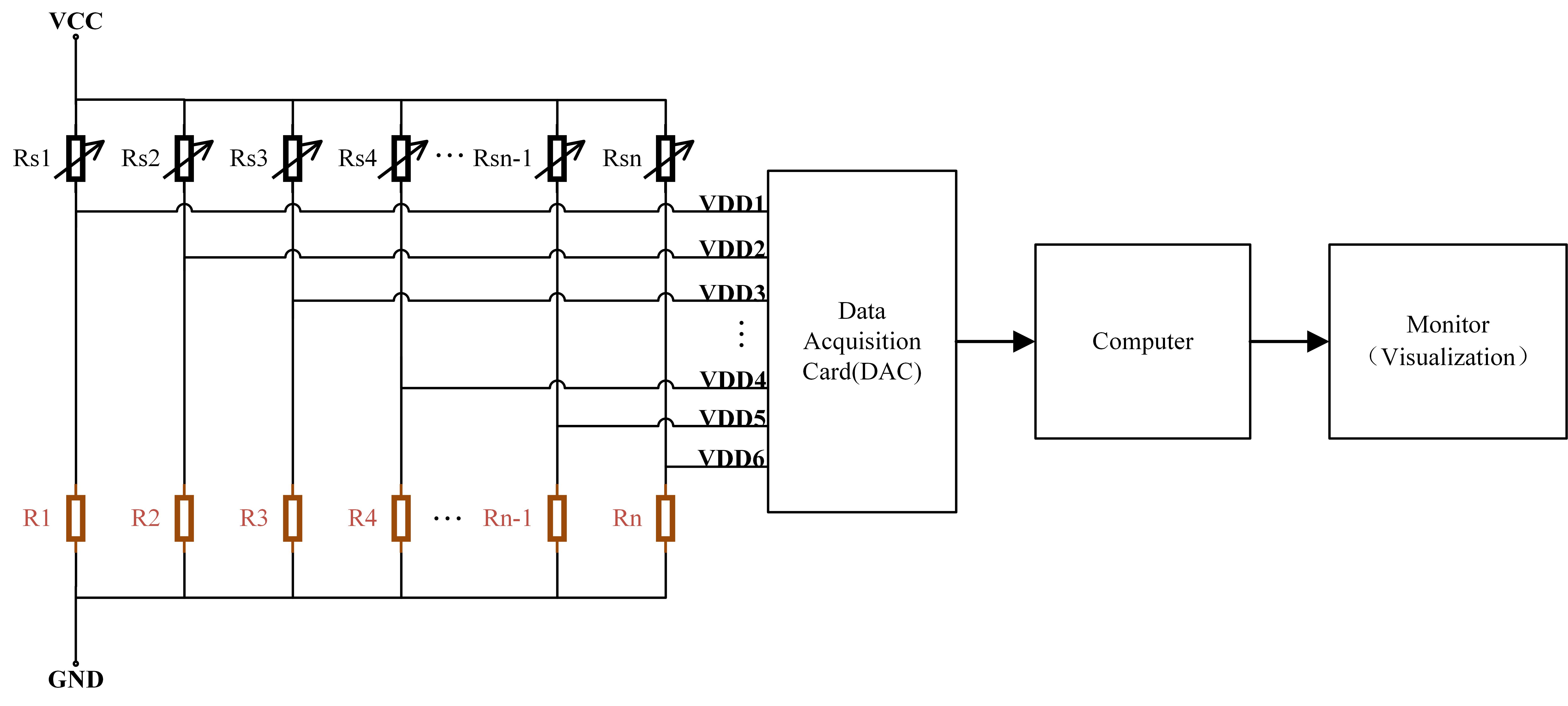


**Figure S15. Schematic diagram of the test system of flexible sensor arrays.** The whole measuring system ensures the testability of signals by the principle of voltage division of resistors close to the resistance value in series. The testing voltage is collected and processed in the data acquisition card, and finally transmitted to the upper computer for output and display.


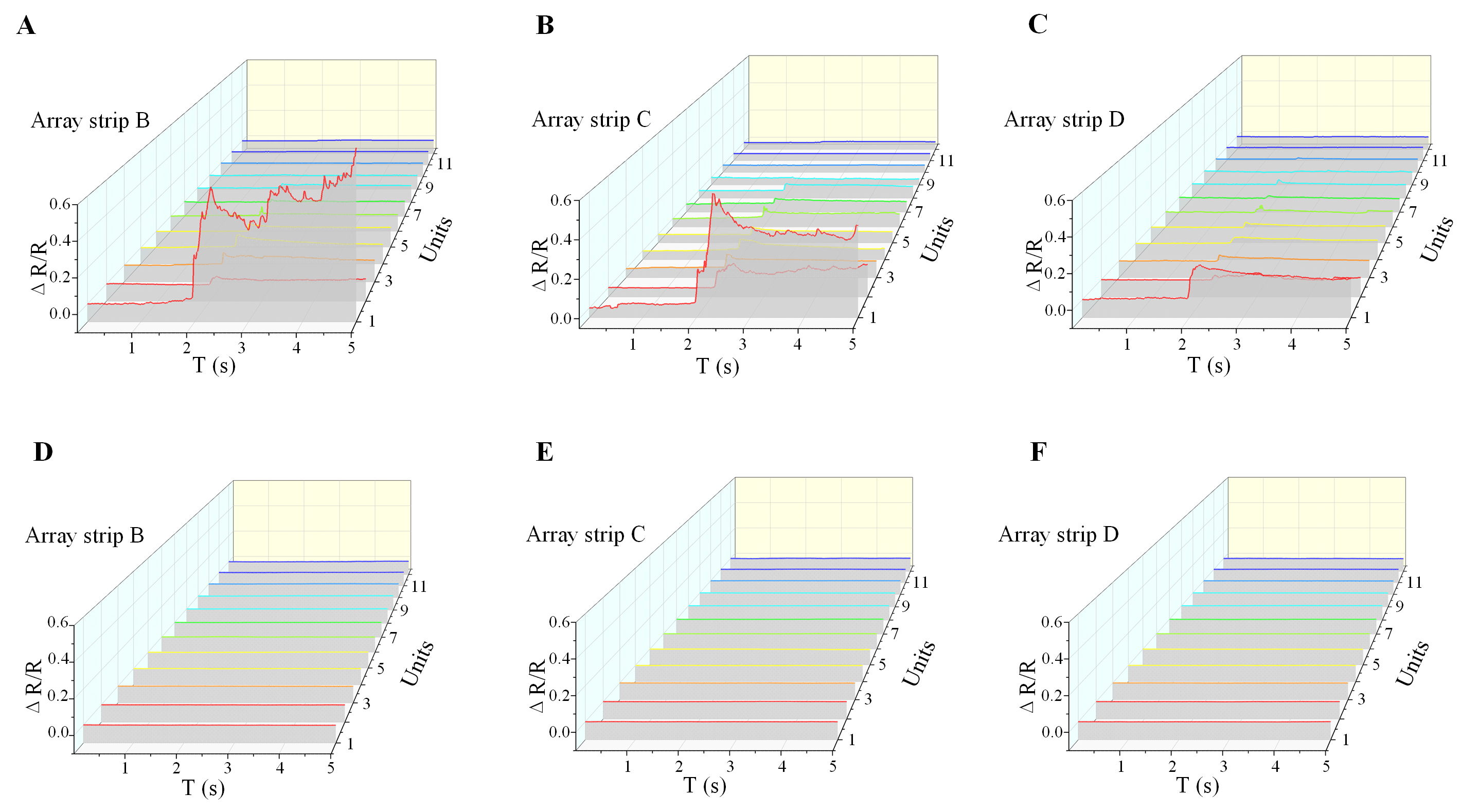


**Figure S16. Characteristic signals of other sensor array strips during the intubation test.** (A) - (C) Step response of each sensor on the sensor array strip B, C, and D when the intubation guide wire is inserted into the esophagus. (D) - (F) No response of each sensor on the sensor array strip B, C, and D when the intubation guide wire is inserted into the trachea.

**Table S1.** **References of elasticity modulus of different substrates.**

| **Materials** | **Elasticity modulus (MPa)** |
| --- | --- |
| Alginate hydrogel  Hybrid gels of alginate and polyacrylamide | 0.017  0.029 |
| Ecoflex | 0.0689 |
| PDMS | 0.78 |
| EVA (ethylene-vinyl acetate copolymer) | 7.9 |
| NOA | 137.9 |
| Structured steel | 2.5×10^5^ |

Seven representative materials with different elasticity modulus^[1-3]^ are designed as the structured substrate respectively. Strain simulation of different material combinations is used to investigate the factors affecting the crosstalk-free ability of the sensor array.

**References**

[1] B. Wang, E. Engay, P. R. Stubbe, S. Z. Moghaddam, E. Thormann, K. Almdal, A. Islam, Y. Yang, *Nat. Commun.* **2022**, *13*.

[2] S. Choi, S. I. Han, D. Kim, T. Hyeon, D.-H. Kim, *Chem. Soc. Rev.* **2019**, *48*, 1566.

[3] J. Peng, G. J. Snyder, *Science* **2019**, *366*, 690.
